# Type I interferon signaling is required for resistance to primary influenza virus infection and vaccine-induced long-term immunity

**DOI:** 10.1128/jvi.00229-26

**Published:** 2026-03-27

**Authors:** Ki-Hye Kim, Hye Suk Hwang, Surya Sekhar Pal, Chau Thuy Tien Le, Phillip Grovenstein, Mahmuda Yeasmin, Jae Min Song, Young-Man Kwon, Baozhong Wang, Sang-Moo Kang

**Affiliations:** 1 Center for Inflammation, Immunity and Infection, Institute for Biomedical Sciences, Georgia State University 1373, Atlanta, Georgia, USA; 2 Department of Biomedical Science, Sunchon National University 65380, Suncheon, South Korea; 3 Department of Next Generation Applied Sciences, Graduate School, Sungshin Women’s University, Seoul, South Korea; 4 Department of Pathobiological Sciences, School of Veterinary Medicine, Louisiana State University 5779, Baton Rouge, Louisiana, USA; St Jude Children's Research Hospital, Memphis, Tennessee, USA

**Keywords:** type I IFN receptor, influenza vaccine, neutrophils, durable immunity

## Abstract

Type I interferons (IFNs) are critical for early antiviral defense, yet their role in shaping vaccine-induced immunity remains incompletely understood. Here, we investigated the impact of type I IFN receptor (IFNαβR) deficiency on immune responses to influenza virus infection and vaccination in IFNαβR-deficient (AB6) mice. AB6 mice exhibited profound susceptibility to influenza virus infection, characterized by accelerated weight loss, elevated lung viral loads, and severe histopathology. Despite inducing short-term IgG antibodies and protection against weight loss after H5 hemagglutinin virus-like particle (H5 VLP) vaccination, AB6 mice were less effective in controlling lung viral loads and inflammation after lethal influenza virus infection, compared to the wild-type (B6) mice. IFNαβR deficiency dysregulated chemokines and numerous innate immune cells, particularly neutrophils, contributing to lung pathology after H5 VLP vaccination and influenza virus infection. AB6 mice exhibited a faster kinetics of waning IgG antibodies and lower efficacy of long-term protection after vaccination than wild-type B6 mice.

**IMPORTANCE:**

Type I interferons (IFNs) are essential mediators of antiviral defense, but their contribution to vaccine-induced immunity remains unclear. This study reveals that type I IFN receptor signaling is dispensable for the initial antibody induction but is critical for sustaining long-term humoral immunity and balanced immune regulation after influenza vaccination and infection. Loss of IFNαβR signaling leads to impaired viral control, excessive neutrophil-driven inflammation, and disrupted immune cell homeostasis. These findings highlight type I IFN signaling as a key integrator of innate and adaptive immune responses required for adequate and durable antiviral protection.

## INTRODUCTION

The induction of virus-specific neutralizing antibodies through vaccination is the primary strategy for conferring protective immunity against influenza (1). Most licensed influenza vaccines aim to generate robust humoral responses; however, the innate immune signals that shape vaccine efficacy, particularly in the context of long-term immunity and immunopathology, remain incompletely understood. Type I interferons (IFNαβ) are key cytokines rapidly produced in response to viral infections and are critical for initiating antiviral defenses. By signaling through the type I IFNαβ receptor (IFNαβR), they induce a broad range of interferon-stimulated genes that restrict viral replication, enhance antigen presentation, and modulate immune cell activation and trafficking (2). In the context of influenza virus infection, IFNαβR signaling has been shown to be essential for early viral clearance, survival, and orchestration of both innate and adaptive immune responses (2–4). Additionally, type I IFNs regulate the localization and function of dendritic cells (DCs), T cell priming, and B cell isotype switching, underscoring their broad immunoregulatory roles (5–7).

Whether type I IFN signaling is required for vaccine-induced immunity remains an area of ongoing research. IFNαR signaling enhances virus-specific IgG2c and IgA antibodies after infection with a live-attenuated influenza virus (8). Exogenous IFNα differentially promotes IgG isotypes (IgG1, IgG2c) and IgA antibodies to influenza M2e protein vaccine after intranasal or intraperitoneal immunization (8). Type I IFN was shown to stimulate the production of IgG subclasses and immunological memory to a soluble protein (6). IFNαβR knockout (IFNαβR^-/-^) mice are often used as a small animal model to test the efficacy of vaccines against Crimean-Congo hemorrhagic fever virus (CCHFV). The CCHFV M-segment DNA vaccination, three times by muscle electrophoresis, induced CCHFV-specific humoral immune responses with neutralizing activity and survival protection against lethal challenge in IFNαβR^-/-^ mice (9). Replicon particle vaccination was reported to induce non-neutralizing anti-nucleoprotein antibody-mediated protection against CCHFV in IFNαβR^-/-^ mice at 4 weeks after immunization (10). Also, CCHFV NP mRNA or glycoprotein mRNA vaccines could induce protective immune responses in IFNαβR^-/-^ mice up to 5 weeks after vaccination (11). These studies suggest that immunogenic vaccine antigens can elicit protective immune responses even in the absence of type I IFN signaling. In addition, excessive or dysregulated type I IFN responses have been implicated in promoting immunopathology during respiratory viral infections, including influenza, by enhancing inflammatory chemokine production and driving tissue damage (12, 13). However, the roles of type I IFN signaling in regulating innate immune responses and maintaining long-lived IgG antibody responses after vaccination and infection largely remain unknown.

Virus-like particle (VLP)-based vaccines have emerged as a platform that mimics the structure and antigenic properties of viruses while ensuring safety. Hemagglutinin (HA)-based VLP vaccines present HA in its native trimeric conformation and retain hemagglutination activity, thereby eliciting potent HA-specific immune responses (14, 15). Moreover, VLP vaccines targeting pandemic influenza strains such as H5N1, H7N9, and 2009 H1N1 have demonstrated acceptable safety and immunogenicity profiles in human trials, supporting their potential as a next-generation influenza vaccine platform (16–18).

In this study, we aimed to dissect the role of type I IFN receptor signaling in both influenza virus infection and H5 HA VLP vaccine-induced immunity using IFNαβR-deficient (AB6) mice. Specifically, we investigated how the absence of IFNαβR signaling impacts innate antiviral defense, humoral immune responses, lung immunopathology, and the development and maintenance of short-term and long-term protective immunity. By comparing infection- versus vaccine-induced immune responses in the presence and absence of type I IFN signaling, we sought to delineate the contributions of IFNαβR to shaping innate immune responses, long-term IgG antibody levels, and protection against the influenza virus.

## RESULTS

### Deficiency in type I IFN receptor signaling leads to increased vulnerability to influenza virus infection

To determine the roles of type I IFNαβR signaling in host protection against primary influenza virus infection, naïve C57BL/6 (B6) wild-type (WT) and IFN-α/β receptor-deficient (IFNαβR⁻/⁻; AB6) mice were intranasally infected with sublethal doses of influenza viruses (Fig. S1). Upon infection with a low dose (0.2×LD_50_) of A/Vietnam rgH5N1 virus, AB6 mice exhibited rapid and severe weight loss and succumbed to infection, with 100% mortality observed by day 8 post-infection (Fig. S1A and B). In contrast, B6 mice exhibited moderate weight loss (~12%) and a 100% survival rate (Fig. S1A and B). A similar trend was observed following infection with a sublethal dose (0.3×LD_50_) of A/California/2009 H1N1 virus. AB6 mice exhibited progressive weight loss reaching humane endpoints for euthanasia, while B6 mice demonstrated recovery after transient weight loss, with a survival rate of 75% (Fig. S1C and D). These findings indicate that the absence of type I IFNαβR signaling results in a significantly increased susceptibility to influenza virus infection, highlighting the essential role of IFNαβR-mediated signaling in early antiviral host defense.

### Type I IFN receptor signaling is dispensable for vaccine-induced early humoral immune responses at 2 weeks after vaccination

**Fig 1:**
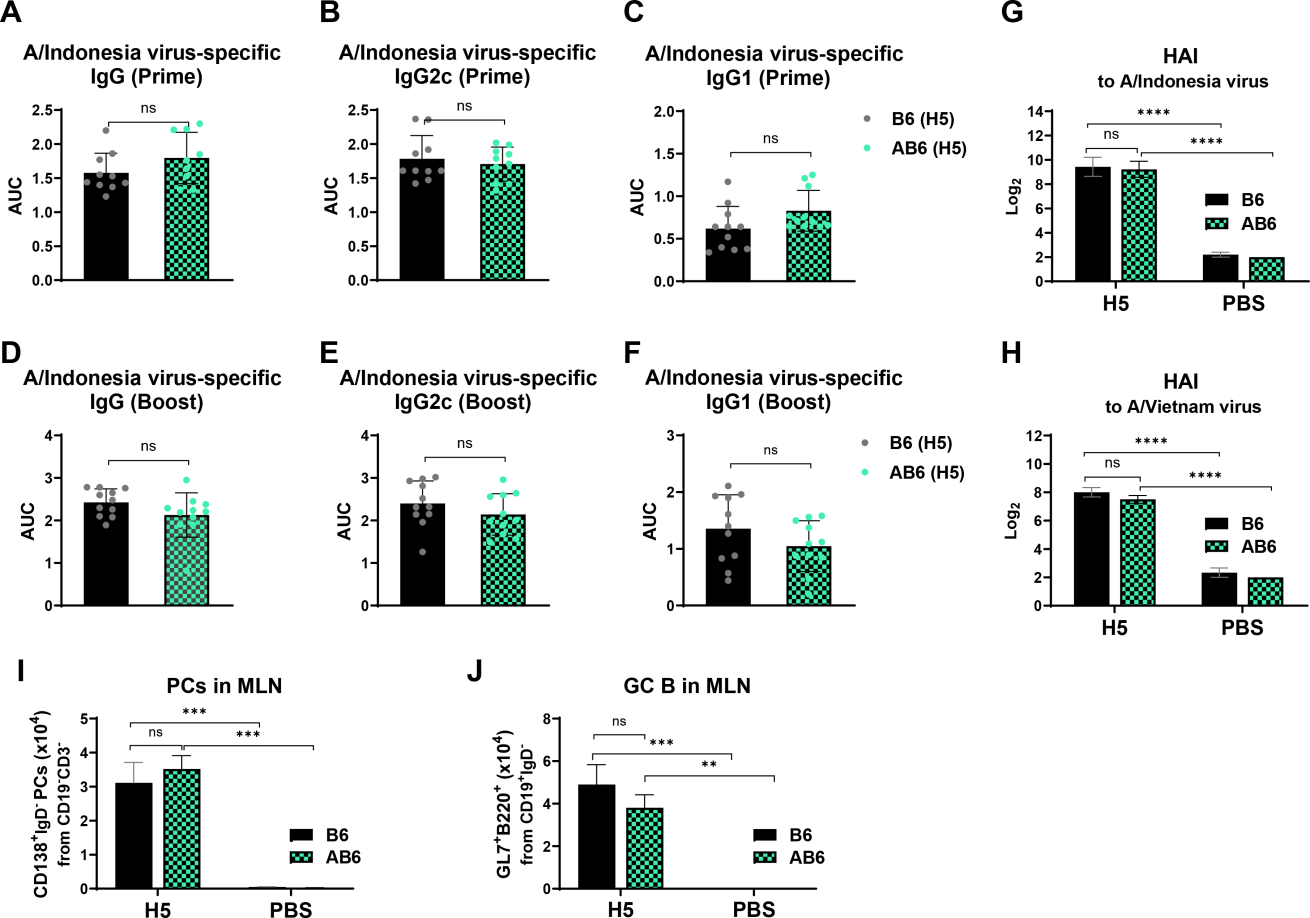
Type I IFNαβR signaling is not required for inducing humoral responses at early time points after H5 HA VLP vaccination. (**A–H**) C57BL/6 (B6) and IFN-α/β receptor-deficient (AB6) mice (*n* = 8–11 mice per group) were intramuscularly immunized with 10 µg of H5 HA VLP (H5) vaccine derived from HA of A/Indonesia/2005 (H5N1) or phosphate-buffered saline (PBS) (no vaccine mock control) at weeks 0 (prime) and 3 (boost). (**A–C**) Serum levels of virus-specific total IgG, IgG2c, and IgG1 following prime vaccination, as presented in AUC values. (**D–F**) Corresponding antibody levels following booster vaccination, as presented in AUC values. (**G, H**) HAI titers against reassortant (rg) H5N1 viruses (homologous A/Indonesia, heterologous A/Vietnam). (**I, J**) Cell numbers of plasma cells (PC, CD138^+^IgD^-^CD19^-^CD3^-^) and germinal center B cells (GC B, GL7^+^B220^+^CD19^+^IgD^-^CD3^-^) in MLNs (mediastinal lymph nodes) from B6 and AB6 mice were analyzed by flow cytometry (Fig. S6) 5 days after challenge (A/Vietnam rgH5N1 virus). Cell number data represent results from two independent experiments with four mice per group (*n* = 4). Data are presented as mean ± standard error of the mean (SEM). Statistical analysis was performed using paired *t*-test (**A–F**) and two-way analysis of variance (ANOVA) with multiple comparisons (**G–I**). **, *P* < 0.01; ***, *P* < 0.001; ****, *P* < 0.0001; ns, not significant.

To assess whether IFNαβR signaling is essential for the induction of protective humoral immunity, C57BL/6 WT (B6) and IFNαβR^-/-^ (AB6) mice were intramuscularly immunized with 10 µg of H5 HA VLP (H5) vaccine derived from A/Indonesia/2005 at weeks 0 and 3. At 2 weeks following prime immunization, A/Indonesia virus-specific IgG antibodies were similarly induced to high levels in the sera of both B6 and AB6 mice (Fig. 1A), as reflected by comparable area under the curve (AUC) values (B6: 1.6 vs AB6: 1.8). The levels of virus-specific IgG2c and IgG1 antibodies were comparable between B6 and AB6 groups, with IgG2c antibodies showing higher responses (AUC 1.7) than IgG1 (AUC 0.7) in both groups (Fig. 1B and C). At 2 weeks after boost immunization (Fig. 1D through F), both B6 and AB6 mice further increased virus-specific antibody responses. Total IgG levels increased to higher AUC values (B6: 2.43 vs AB6: 2.13) in both groups (Fig. 1D). Similarly, IgG2c responses remained higher than IgG1 responses following boost, with IgG2c AUC values of 2.40 and 2.16, and IgG1 AUC values of 1.35 and 1.0 in B6 and AB6 mice, respectively (Fig. 1D through F), promoting Th1-type IgG2c antibody responses even under the deficiency of type I IFN receptor signaling. To further examine the functionality of humoral responses, hemagglutination inhibition (HAI) assays were performed from 2 week boost sera. HAI titers against rgH5N1 viruses were detected at comparable levels (512 titers against homologous A/Indonesia; 256 titers against heterologous A/Vietnam) in B6 and AB6 mice following immunization (Fig. 1G and H), indicating the generation of protective levels of HAI activity antibodies even in the IFNαβ receptor signaling-deficient status. Moreover, vaccine-induced plasma cells (PCs) and germinal center B cells were generated at comparable levels in B6 and AB6 mice, showing a slight increase in PC and a modest reduction in GC B cells from AB6 mice, without statistical significance (Fig. 1I and J). Collectively, these results suggest that type I IFNαβR signaling is not required for the effective generation of vaccine-induced IgG antibody responses at early time points.

### AB6 mice display lower efficacy in viral clearance and prevention of lung inflammation after virus infection

**Fig 2:**
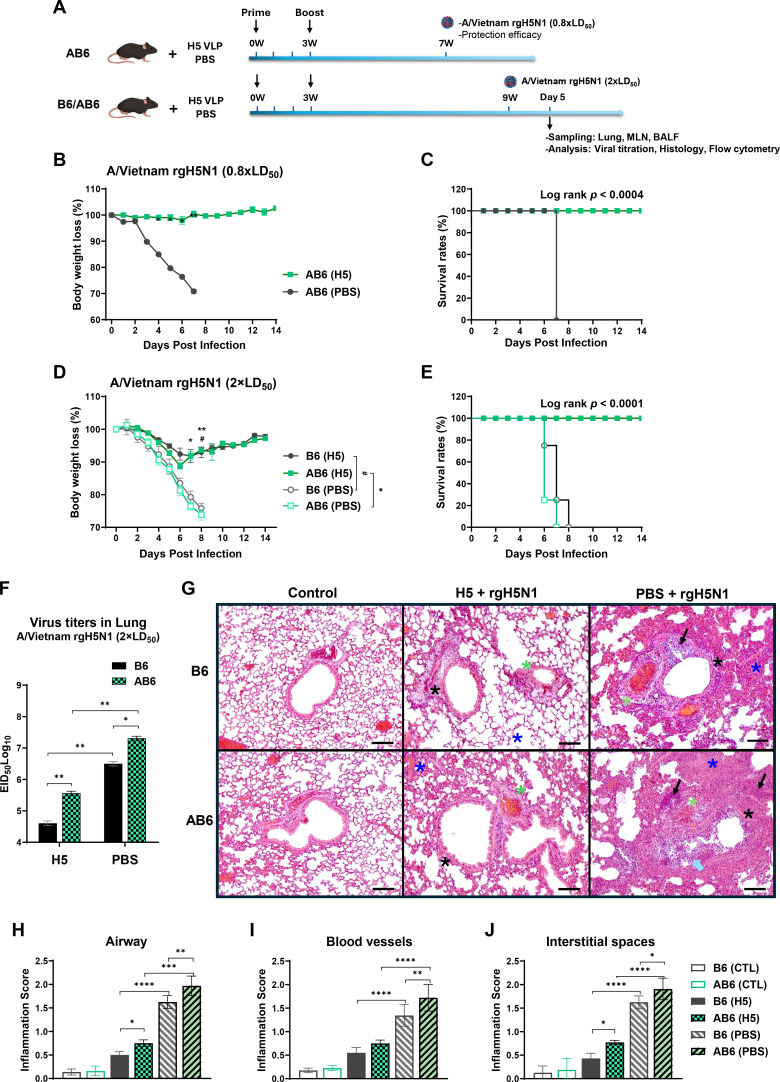
H5 VLP vaccination provides adequate short- and mid-term protection against heterologous H5N1 challenge in the absence of IFNαβR signaling. (**A**) Schematic diagram of immunization and viral challenge experiments. Vaccinated AB6 mice (top, *n* = 4 mice per group) received two doses of H5 VLP or PBS at week 0 (prime) and 3 (boost), followed by challenge with a sublethal dose (0.8×LD₅₀) of heterologous A/Vietnam rgH5N1 virus at week 7 to assess protection efficacy. Both C57BL/6 (B6) and AB6 mice (bottom, *n* = 4–5 mice per group) were immunized similarly and challenged with a lethal dose (2×LD₅₀) of the same virus at week 9. Samples from the lungs, MLN, and bronchoalveolar lavage fluid (BALF) were collected at 5 dpi for viral titration, histological analysis, and flow cytometry. (**B and C**) Body weight and survival of AB6 mice after challenge with a sublethal dose (0.8×LD₅₀) of A/Vietnam rgH5N1 virus at 4 weeks post-boost (*n* = 4 mice per group). (**D and E**) Body weight changes and survival of B6 and AB6 mice after challenge with a lethal dose (2×LD₅₀) A/Vietnam rgH5N1 virus at 6 weeks post-boost (*n* = 5 mice per group). (**F**) Lung viral titers were measured on day 5 p.i. in vaccinated and naïve B6 and AB6 mice using the 50% egg infectious dose (EID_50_) assay. (**G**) Representative microphotographs of hematoxylin and eosin (H&E)-stained lung sections on day 5 p.i. Inflammatory foci are observed in the airways (black asterisk), blood vessels (green asterisk), and interstitial spaces (blue asterisk). Severe distortion of airway structure and hyperplasia of pneumocytes are indicated by open arrows (blue), while solid arrows denote infiltration of inflammatory cells. Arrowheads indicate interstitial edema. Scale bars represent 100 µm. (**H–J**) Histopathological scoring of inflammation severity around the airway, blood vessels, and interstitial spaces. Data are presented as mean ± SEM. Statistical analysis was performed using two-way ANOVA with multiple comparisons (**B, D, F, H–J**) and survival curve comparison with the log-rank (Mantel-Cox) test (**C, E**). *^,#^, *, *P* < 0.05; **, *P* < 0.01; ***, *P* < 0.001; ****, *P* < 0.0001.

To assess the short-lived protective efficacy, mice were challenged with either a low lethal dose (0.8×LD_50_) or a high lethal dose (2×LD_50_) of heterologous A/Vietnam rgH5N1 virus at 4 or 6 weeks after boost immunization (Fig. 2A). Under low-dose viral challenge, all vaccinated AB6 mice were fully protected and showed no weight loss throughout the course of infection, whereas all naïve AB6 mice succumbed to death (Fig. 2B and C). The difference in survival between vaccinated and naïve mice was statistically significant (log-rank test, *P* < 0.0004). Upon challenge with a high lethal dose, the B6-vaccinated group exhibited moderate and transient weight loss (~8.2%; Fig. 2D and E), whereas the vaccinated AB6 group showed slightly greater weight loss (~11.2% on day 5 post-challenge; Fig. 2D and E). Both vaccinated B6 and AB6 mice subsequently recovered. In contrast, unvaccinated (PBS) B6 and AB6 mice experienced rapid and severe weight loss, resulting in 0% survival. The difference in survival between vaccinated and unvaccinated groups was highly significant (log-rank test, *P* < 0.0001, Fig. 2D and E).

Following a high-dose viral challenge, viral titers in lung tissues collected on 5 days post-infection (dpi) were highest in naïve AB6 mice (PBS), reaching 2.0×10⁷ EID_50_ per lung, which was approximately 6.3-fold higher than those observed in naïve B6 mice (0.3×10⁷ EID_50_ per lung; *P* < 0.05) (Fig. 2F). This indicates a statistically significant difference in viral replication between the two mouse strains. In contrast, vaccinated B6 mice showed significantly lower lung viral titers (4×10^4^ EID_50_ per lung) compared to vaccinated AB6 mice (3.7×10^5^ EID_50_ per lung), equivalent to an approximately ninefold reduction (*P* < 0.01) (Fig. 2F). Importantly, both vaccinated groups exhibited markedly reduced viral loads relative to their unvaccinated control, with B6 and AB6 mice displaying ~78-fold and ~55-fold reductions, respectively. The lung viral titers indicate vaccine-mediated protection in both strains compared to their respective PBS control groups. On 5 dpi with A/Vietnam rgH5N1 virus, histopathological examination of lung tissues revealed severe pulmonary pathology in unvaccinated B6 and AB6 mice (Fig. 2G). H&E-stained lung sections showed more dense infiltration of immune cells around the airways (peribronchial, Fig. 2G and H), blood vessels (perivascular, Fig. 2G and I), and interstitial spaces (Fig. 2G and J) from AB6 mice than B6 mice, accompanied by pronounced alveolar edema and marked thickening of the interstitial septa. These pathological features were associated with significant distortion of airway structures and hyperplasia of pneumocytes. Representative microphotographs highlight inflammatory foci within the airways (black asterisk), blood vessels (green asterisk), and interstitial spaces (blue asterisk). Compared to the PBS + rgH5N1 group, vaccinated H5 + rgH5N1 mice exhibited minimal histopathological alterations, with largely preserved lung architecture and a substantial reduction in inflammatory cell infiltration. AB6 vaccinated mice displayed moderately higher inflammation scores in the airways (Fig. 2H) and interstitial spaces (Fig. 2J) than B6 vaccinated mice, consistent with the representative histology images (Fig. 2G). These findings collectively demonstrate that, compared with B6 mice, AB6 mice deficient in IFNαβ receptor signaling exhibit impaired viral clearance and reduced capacity to prevent lung inflammation after virus infection, with or without vaccination.

### Loss of type I IFNαβR signaling triggers infiltration of NK and NKT cells into the lung during influenza virus infection

**Fig 3:**
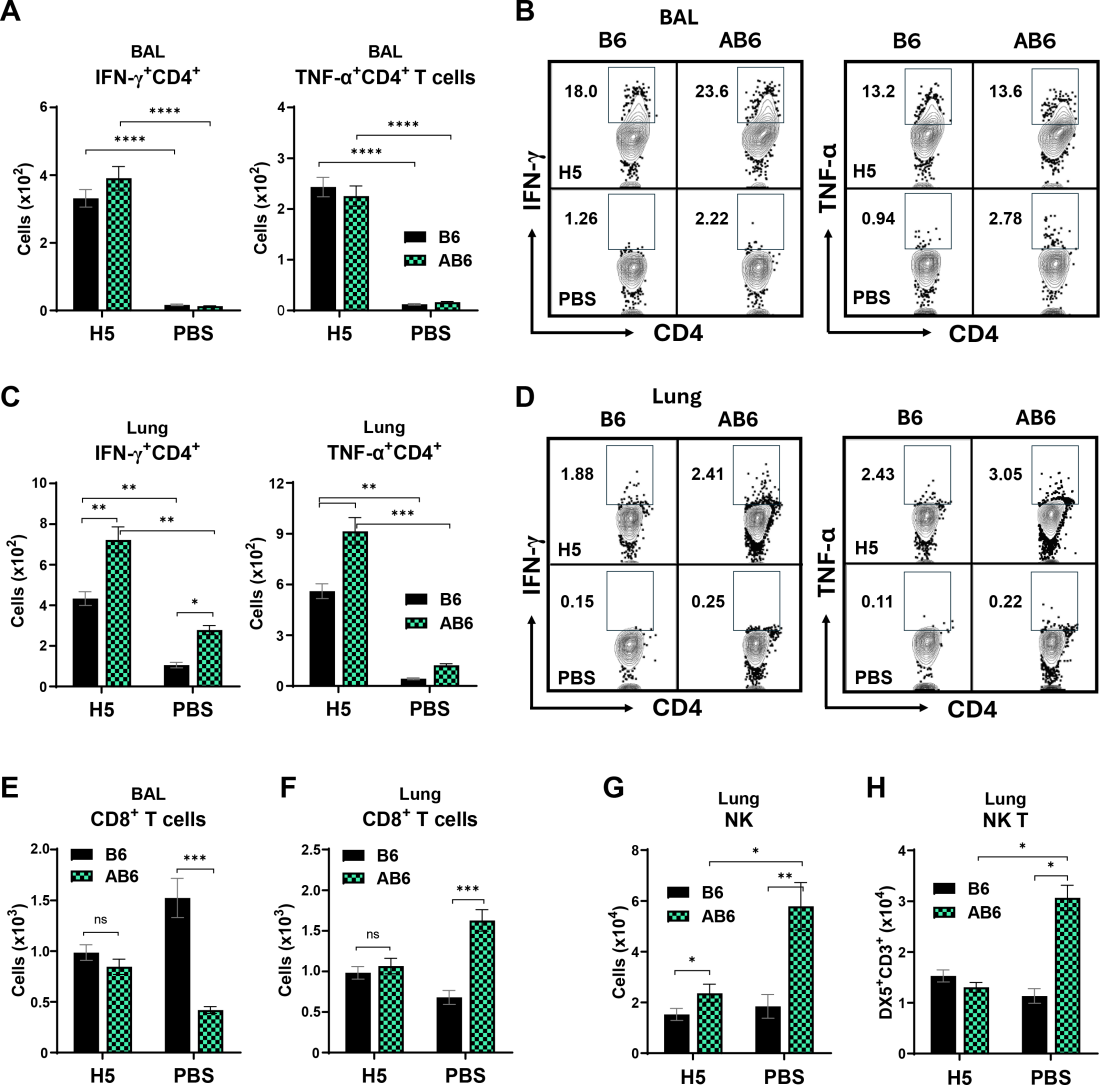
Differential levels of effector T cells, NK, and NKT cells in the airways and lung tissues after influenza virus infection. (**A–F**) Flow cytometric analysis of effector T cell populations in the airway BAL fluids and lung tissues of vaccinated and naïve (PBS) B6 and AB6 mice (*n* = 4–5 mice per group) on day 5 p.i. with a lethal dose (2×LD₅₀) of A/Vietnam rgH5N1 virus. (**A–D**) Numbers and frequencies of IFN-γ^+^CD4^+^ and TNF-α^+^CD4^+^ T cells in BAL and lungs. Numbers within the representative flow plots indicate percentages of effector CD4 T cells out of total CD4 T cells (Fig. S2). (**E and F**) CD8^+^ T cells in BAL and lungs. (**G and H**) Quantification of NK and NKT cell populations in lung tissues of B6 and AB6 mice (*n* = 4–5 mice per group). Data are presented as mean ± SEM. Statistical significance was determined using two-way ANOVA with Tukey’s multiple comparisons test. *, *P* < 0.05; **, *P* < 0.01; ***, *P* < 0.001; ****, *P* < 0.0001; ns, not significant.

On 5 dpi with A/Vietnam rgH5N1 virus, the numbers of total lymphocytes and CD4**^+^** T cells in the BAL of naïve AB6 mice were decreased compared with those observed in naïve B6 mice (Fig. S2). In contrast, in the lungs, total lymphocytes and CD4^+^ T cells were increased by 1.7- and 2.9-fold, respectively, in AB6 mice relative to those in naïve B6 mice (Fig. S2). In the BAL, the numbers and frequency of IFN-γ^+^CD4^+^ T cells in vaccinated AB6 mice were comparable to (or higher than) those of the vaccinated B6 mice, whereas TNF-α^+^CD4^+^ T cells were observed at similar levels between the AB6 and B6 groups (Fig. 3A and B). Meanwhile, in the lungs, vaccinated AB6 mice exhibited higher numbers of both IFN-γ^+^CD4^+^ and TNF-α^+^CD4^+^ T cells compared to vaccinated B6 mice (Fig. 3C and D). In contrast, the number of CD8^+^ T cells was markedly elevated in the BAL of B6 (PBS) mice and in the lungs of AB6 (PBS) (Fig. 3E and F). A similarly low level of CD8^+^ T cells was observed in the BAL of vaccinated B6 and lungs of vaccinated AB6 (Fig. 3E and F). Moreover, increased infiltration of NK cells was observed in the lungs of both naïve (PBS) and vaccinated AB6 (H5) mice, and NKT cells in the naïve AB6 control compared to their B6 counterparts (Fig. 3G and H), possibly correlating with higher lung viral titers and inflammation in naïve mice after viral infection, which could be reduced by H5 HA VLP vaccination. These findings suggest that the absence of type I IFN signaling promotes the induction of effector CD4 T cells in the lung following vaccination and infection and the recruitment of innate NK and NKT cells into the lung, correlating with high viral loads from unvaccinated AB6 mice after infection.

### Loss of type I IFNαβR signaling leads to innate immune dysregulation with increased neutrophil infiltration after influenza virus infection

**Fig 4:**
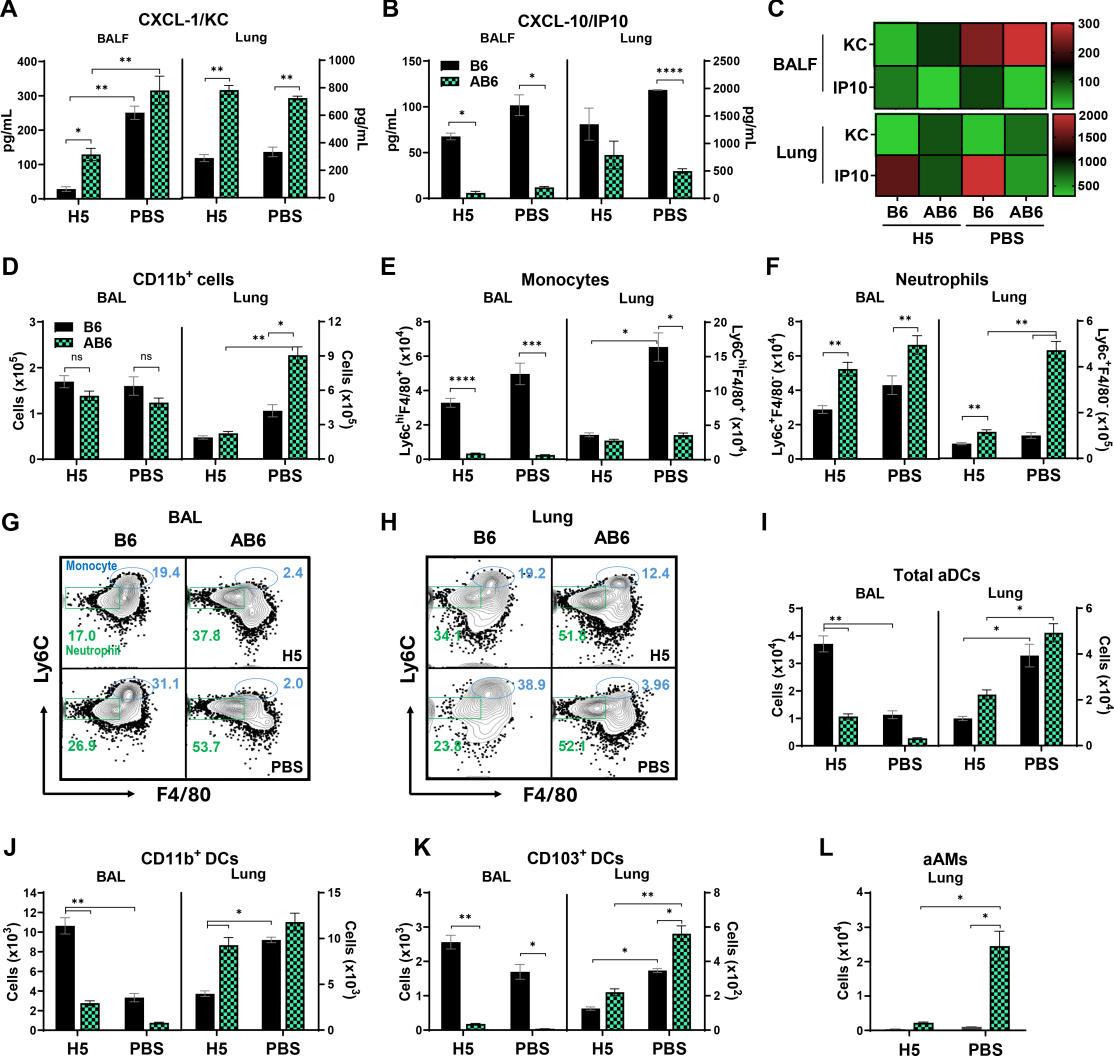
Dysregulated chemokine production and enhanced innate immune cell recruitment to the lung in AB6 mice after influenza virus infection. (**A–C**) Levels of CXCL-1/KC and CXCL-10/IP-10 chemokines were measured by ELISA in BALF and lung homogenates of B6 and AB6 mice (*n* = 4–5 mice per group) on day 5 p.i. with A/Vietnam rgH5N1 virus. (**C**) Heat map representing mean concentrations of individual chemokines in BALF and lung tissues. (**D–H**) Flow cytometric quantification of CD11b^+^ myeloid cells, monocytes (Ly6c^hi^F4/80^+^CD11b^+^), and neutrophils (Ly6c^+^F4/80^-^CD11b^+^) in BAL and lung tissues (*n* = 4–5 mice per group), respectively. (**G and H**) Numbers in the representative flow plots indicate percentages of monocytes and neutrophils, as described in the gating strategy (Fig. S7). (**I–K**) Numbers of activated dendritic cells (aDCs; CD11c^+^MHCII^+^), including CD11b^+^ (CD11b^+^CD11c^+^MHCII^+^CD103^-^) and CD103^+^ (CD103^+^CD11c^+^MHCII^+^CD11b^-^) subsets, in BAL and lungs. (**L**) Activated alveolar macrophages (aAMs; MHCII^+^CD11c^+^F4/80^+^) in the lungs. Data (*n* = 4–5 mice per group) are presented as mean ± SEM. Statistical analysis was performed using two-way ANOVA with Tukey’s multiple comparisons test. *, *P* < 0.05; **, *P* < 0.01;***, *P* < 0.001; ****, *P* < 0.0001; ns, not significant.

To further investigate the innate immune mechanisms underlying vaccine-induced protection or immunopathology, we analyzed chemokine production and innate immune cell recruitment in BAL fluid and lung tissues at 5 dpi with A/Vietnam rgH5N1 virus. Vaccinated and unvaccinated AB6 mice displayed significantly elevated levels of the neutrophil-attracting chemokine CXCL-1/KC in both BAL fluid and lung homogenates compared to their B6 counterparts, with the increase being most pronounced in unvaccinated AB6 mice (Fig. 4A). In contrast, the expression of CXCL-10/IP-10, a chemokine involved in monocyte recruitment, was markedly lower in AB6 mice relative to B6 mice in both compartments, irrespective of vaccination status (Fig. 4B). The heat map provides an overview of the relative chemokine levels in B6 and AB6 mice (Fig. 4C), suggesting an imbalance in chemokine responses associated with type I IFN signaling deficiency.

The elevated CXCL-1/KC levels observed in AB6 mice might be associated with a significant increase in CD11b^+^ myeloid cells in the lung (PBS control) but not in BAL (Fig. 4D). In addition, monocyte levels were significantly reduced in AB6 mice compared to B6 mice, irrespective of vaccination status in the BAL and lung (Fig. 4E). Consistent with the altered chemokine milieu, a pronounced infiltration of neutrophils was observed in both the BAL and lungs of AB6 mice (PBS control) than B6 mice (Fig. 4F through H). Notably, vaccinated B6 mice showed markedly reduced neutrophil accumulation in both the BAL and lung tissue compared to both vaccinated and PBS control AB6 groups (Fig. 4F through H). Representative flow plots further support the differential infiltration of monocytes (Ly6c^hi^F4/80^+^CD11b^+^) and neutrophils (Ly6c^+^F4/80^-^CD11b^+^) into BAL and lung tissues (Fig. 4G and H).

Flow cytometric analysis revealed that total aDCs (CD11c^+^MHCII^+^), including both CD11b^+^ and CD103^+^ subsets, were significantly reduced in the BAL of AB6 mice compared to B6 controls (Fig. 4I through K). Interestingly, in lung tissues, both vaccinated and unvaccinated AB6 mice exhibited increased infiltration of aDCs and CD11b^+^, CD103^+^ DC subsets (Fig. 4I through K), likely reflecting a compensatory or dysregulated response driven by unresolved viral replication and inflammation. The infiltration of aAMs closely paralleled the neutrophil dynamics, showing significantly increased numbers in lung tissues of PBS control AB6 mice compared to B6 (Fig. 4L). Overall, recruiting innate immune cells into the airways (BAL) rather than into the lungs might be correlated with protection against the virus in both H5 HA VLP-vaccinated WT and AB6 mice (Fig. 4E through L).

To further investigate the roles of type I IFNR signaling in the acute recruitment of innate immune and antigen-presenting cells (APCs), we analyzed peritoneal exudate cells 1 day after intraperitoneal injection of H5 HA VLP (10 µg) vaccine or PBS (mock) in naïve AB6 and B6 mice, in the absence of viral challenge. Following administration of the vaccine, AB6 mice exhibited significantly increased numbers of CD11b^+^ myeloid cells in the peritoneal cavity compared to both PBS-treated AB6 controls and vaccinated B6 mice (Fig. S3A). Among these innate cell populations, neutrophils were most prominently recruited to the site of injection in AB6 mice, along with a substantial enrichment of eosinophils and MHCII^+^ macrophages (Fig. S3B through D). In contrast, monocyte infiltration was markedly reduced in AB6 mice relative to B6 mice (Fig. S3E), which preferentially recruit monocytes but neither neutrophils nor eosinophils, indicating a shift in the composition of recruited myeloid cells. We next assessed CD11c^+^F4/80⁻ DCs, which were elevated in AB6 mice at 1 day after injection, corresponding with increased frequencies of aDCs (CD11c^+^MHCII^+^) and CD11b^+^ DCs in AB6 mice (Fig. S3F through H). Collectively, these results indicate that type I IFNR signaling regulates early innate immune responses to vaccination by shaping the recruitment profile of granulocytes, monocytes, and DC precursors at the site of antigen exposure.

### Targeted neutrophil depletion mitigates influenza-induced pathology in type I IFNαβR-deficient mice

**Fig 5:**
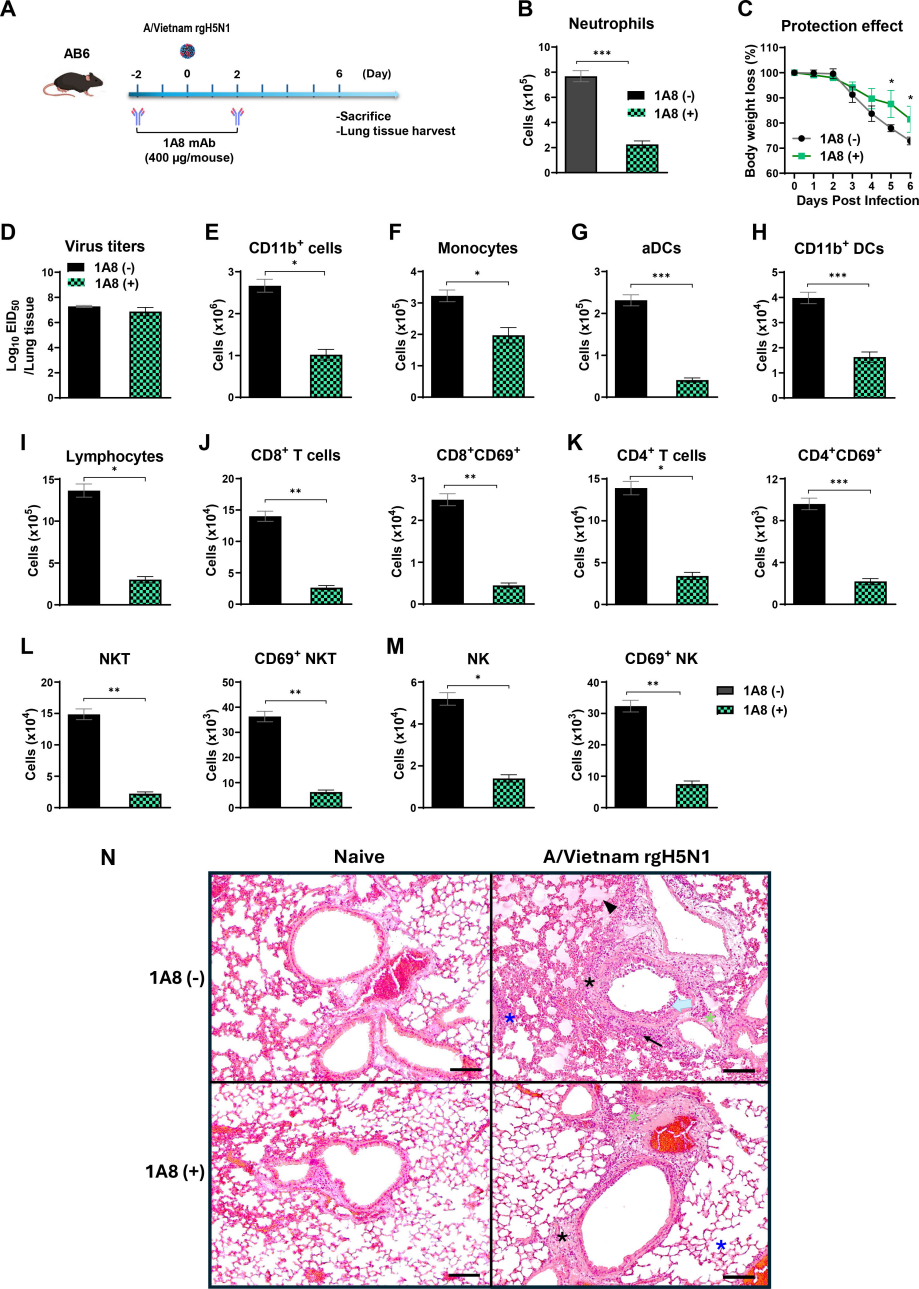
Neutrophil depletion reduces weight loss and lung histopathology, with diminished infiltration of innate immune cells in AB6 mice after influenza virus infection. (**A**) Schematic of the neutrophil depletion experiment. AB6 mice (*n* = 4 mice per group) were treated with anti-Ly6G (1A8) mAb at days −2 and +2 p.i. to deplete neutrophils during A/Vietnam rgH5N1 virus infection. (**B**) Efficacy of neutrophil depletion assessed by flow cytometry. (**C and D**) Body weight change and lung viral titers at day 6 p.i. (**E–M**) Flow cytometric analysis of lung immune cells showing changes in: (**E**) CD11b^+^ myeloid cells, (**F**) monocytes (Ly6c^hi^F4/80^+^CD11b^+^), (**G**) activated DCs (CD11c^+^MHCII^+^CD45^+^F4/80^-^), (**H**) CD11b^+^ DCs (CD11b^+^CD11c^+^MHCII^+^), (**I**) total lymphocytes (CD11b^-^), (**J**) CD8^+^ T cells and activated CD8 T cells (CD8^+^CD69^+^), (**K**) CD4^+^ T cells and activated CD4 T cells (CD4^+^CD69^+^), (**L**) NKT cells (CD49b^+^CD3^+^CD11b^-^) and activated NKT cells (CD69^+^CD49b^+^CD3^+^CD11b^-^), and (**M**) NK cells (CD49b^+^CD3^-^CD11b^-^) and activated NK cells (CD69^+^CD49b^+^CD3^-^CD11b^-^). (**N**) Representative lung histopathology at 6 dpi in AB6 mice treated with or without anti-Ly6G (1A8) mAb, as assessed by H&E staining. Magnification is 100×. Inflammatory foci are observed in the airways (black asterisk), blood vessels (green asterisk), and interstitial spaces (blue asterisk). Severe distortion of airway structure and hyperplasia of pneumocytes are indicated by the open arrow, while the solid arrows denote infiltration of inflammatory cells. Arrowheads indicate interstitial edema. Scale bars represent 100 µm. Data are presented as mean ± SEM. Statistical analysis was performed using a paired *t*-test (**B, D–M**) and two-way ANOVA with multiple comparisons test (**C**). *, *P* < 0.05; **, *P* < 0.01; ***, *P* < 0.001.

To investigate whether reducing neutrophil burden could partially mitigate disease in IFNAR-deficient mice by partially reverting innate immune cell recruitment following influenza virus challenge in AB6 mice, we performed neutrophil depletion using anti-Ly6G (1A8) monoclonal antibody (mAb) at days −2 and +2 relative to Vietnam rgH5N1 infection (Fig. 5A). Flow cytometric analysis confirmed a partial depletion of neutrophils (Fig. 5B), with a reduction of Ly6G^+^ cells (Fig. S4). The 1A8 mAb-treated group showed significantly less weight loss (~18%) compared to the non-treated group (~28%) (Fig. 5C), suggesting a reduction in overall disease severity. Despite this, viral titers in the lungs showed no difference between the 1A8 mAb-treated and non-treated groups (Fig. 5D).

Flow cytometry analysis of innate immune cell populations (Fig. S4) revealed that the numbers of CD11b^+^ myeloid cells and monocytes were reduced in the 1A8 mAb-treated group compared to the control (Fig. 5E and F). Notably, APCs, including aDCs and CD11b^+^ DCs, were significantly reduced in the 1A8 mAb-treated group (Fig. 5G and H). Regarding adaptive immune cells, the numbers of total lymphocytes and T cell subsets, including CD8^+^ T cells, activated CD69^+^CD8^+^ T cells, CD4^+^ T cells, and CD69^+^CD4^+^ T cells, were significantly reduced in the 1A8 mAb-treated group (Fig. 5I through K). Innate lymphocyte populations, including NKT and NK cells, along with their activated (CD69^+^) marker, were significantly decreased in the 1A8 mAb-treated mice (Fig. 5L and M).

Histopathological analysis of lung tissues showed reduced inflammatory cell infiltration and tissue damage in the 1A8 mAb-treated group compared with the untreated AB6 control (Fig. 5N), consistent with lower weight loss changes. These findings suggest that neutrophil depletion improves clinical outcomes in AB6 mice without affecting viral loads, supporting a model of neutrophil-driven immunopathology.

### Type I IFNαβR signaling plays an essential role in sustaining long-term vaccine-induced IgG responses and protection

**Fig 6:**
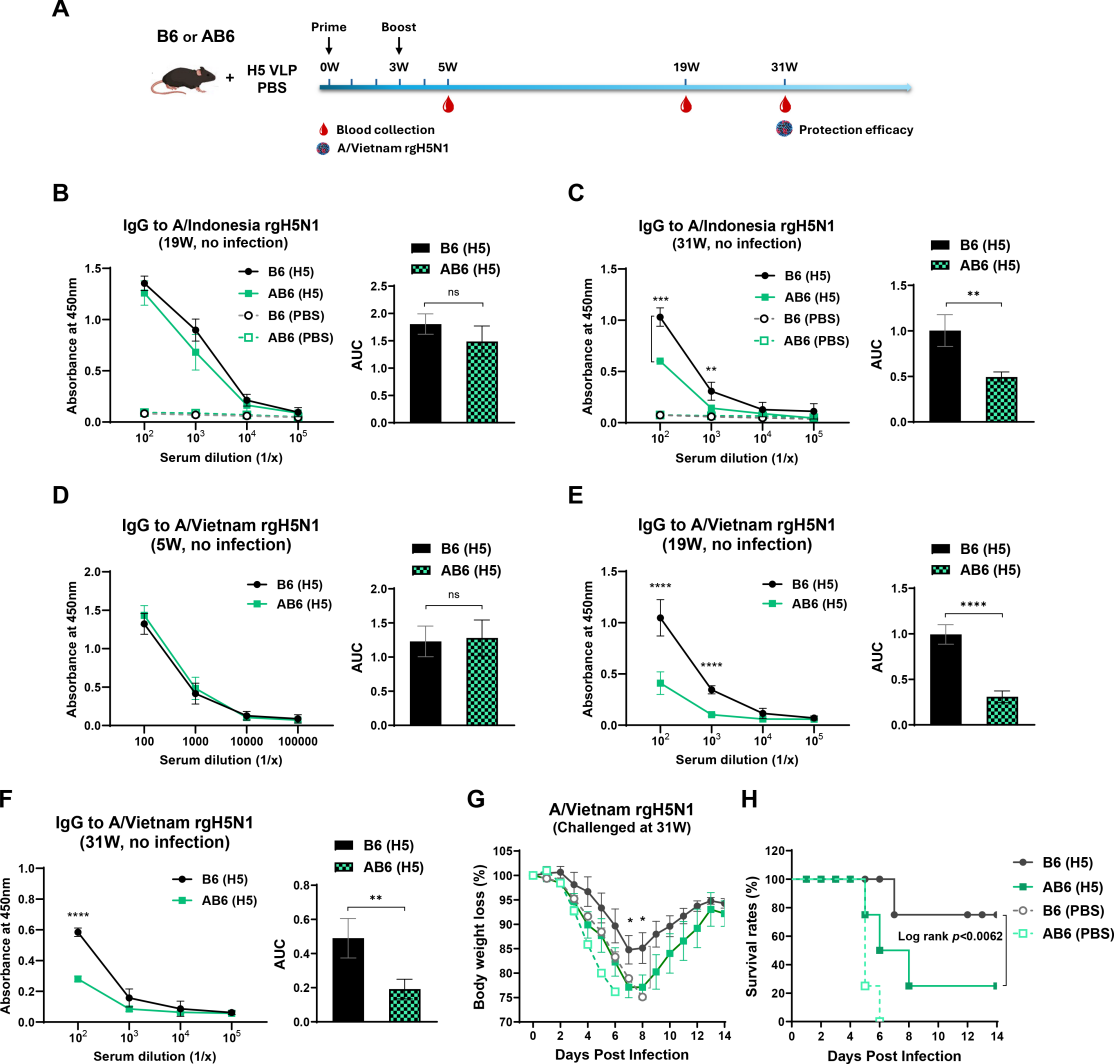
AB6 mice display faster kinetics of waning IgG antibodies and lower cross-protective immunity after vaccination than B6 mice. (**A**) Schematic of immunization and viral challenge experiments (*n* = 4–5 mice per group). (**B and C**) Serum IgG antibodies against the homologous A/Indonesia rgH5N1 virus were measured by ELISA at 19 and 31 weeks. (**D–F**) Serum IgG antibody titers against heterologous A/Vietnam rgH5N1 virus at 5, 19, and 31 weeks after boost vaccination. Left graphs show absorbance at 450 nm versus serum dilution; right graphs show AUC analysis. (**G and H**) Body weight changes and survival following intranasal challenge with a lethal dose of heterologous A/Vietnam rgH5N1 virus at 31 weeks (approximately 7.2 months) after boost. Data are represented as mean ± SEM. Statistical analysis was performed using a two-way ANOVA test and survival curve comparison with the log-rank (Mantel-Cox) test. *, *P* < 0.05; **, *P* < 0.01; ***, *P* < 0.001; ****, *P* < 0.0001; ns, not significant.

To evaluate the role of type I IFNR signaling in the durability of vaccine-induced immunity, B6 and AB6 mice were immunized with H5 HA VLP at weeks 0 and 3. Serum samples were collected at multiple time points (weeks 5, 19, and 31) before virus challenge (Fig. 6A) and at 2 weeks after viral challenge (Fig. S5) for IgG antibody analysis. A/Vietnam rgH5N1 virus challenge was performed at 31 weeks post-boost to evaluate protection, with subsequent monitoring of body weight and survival rates, as outlined in Fig. 6A.

At 19 weeks post-boost, both B6 and AB6 mice immunized with H5 VLP exhibited similarly high levels of IgG antibodies against the homologous A/Indonesia rgH5N1 antigen, as shown in OD readings and AUC values of 1.8 and 1.4, respectively (Fig. 6B). IgG levels for the homologous antigen gradually declined over time, with AUC values declining to 1.0 for B6 and 0.49 for AB6, respectively, at 31 weeks after boost (*P* < 0.01, Fig. 6C). Notably, B6 mice retained significantly higher IgG levels compared to AB6 mice at the 31 week time point (Fig. 6C). IgG responses against the heterologous A/Vietnam rgH5N1 antigen were also assessed at 5, 19, and 31 weeks after boost. At week 5, both B6 and AB6 mice exhibited comparable IgG antibody levels for A/Vietnam H5N1 antigen, with AUC values of approximately 1.2 for both strains (Fig. 6D). However, by week 19 (Fig. 6E), B6 mice displayed significantly higher IgG levels than AB6 mice, with AUC values of 1.0 and 0.3, respectively (*P* < 0.0001, Fig. 6E). This difference remained evident at week 31, although IgG levels in both B6 and AB6 mice had substantially reduced over time, with AUC values declining to 0.49 for B6 and 0.19 for AB6 (*P* < 0.01, Fig. 6F).

To further investigate the durability and evolution of the humoral immune response after viral challenge, serum IgG levels against A/Vietnam and A/Indonesia rgH5N1 antigens were assessed at 2 weeks after A/Vietnam rgH5N1 virus challenge (Fig. S5). Both vaccinated B6 and AB6 mice showed comparable levels of IgG specific to both viral antigens in post-challenge sera. Furthermore, naive (PBS) B6 and AB6 mice elicited comparable levels of IgG antibody responses to both viral antigens in sera collected at 2 weeks after infection with A/Vietnam rgH5N1 virus. This supports the notion that type I IFNR signaling is not strictly required for the initiation and generation of IgG antibody responses at early time points after influenza virus infection or H5 VLP vaccination.

To assess whether these IgG antibody dynamics translated into protective efficacy, mice were monitored for body weight loss following challenge with a lethal dose of heterologous A/Vietnam rgH5N1 virus at 31 weeks after boost vaccination. Unvaccinated mice (PBS) from both strains exhibited severe weight loss and 0% survival (Fig. 6G and H). Vaccinated AB6 mice also showed severe mortality, with a mean peak weight loss of approximately 23% (*P* < 0.05) and a survival rate of 25% (log rank, *P* < 0.0062), compared to vaccinated B6 mice. In contrast, vaccinated B6 mice experienced moderate weight loss (approximately 15%) and a significantly higher survival rate of 75% (Fig. 6G and H). These findings indicate that type I IFNR signaling is critical for the long-term maintenance of cross-reactive IgG antibodies and for achieving protective immunity following vaccination and viral challenge.

## DISCUSSION

**Fig 7:**
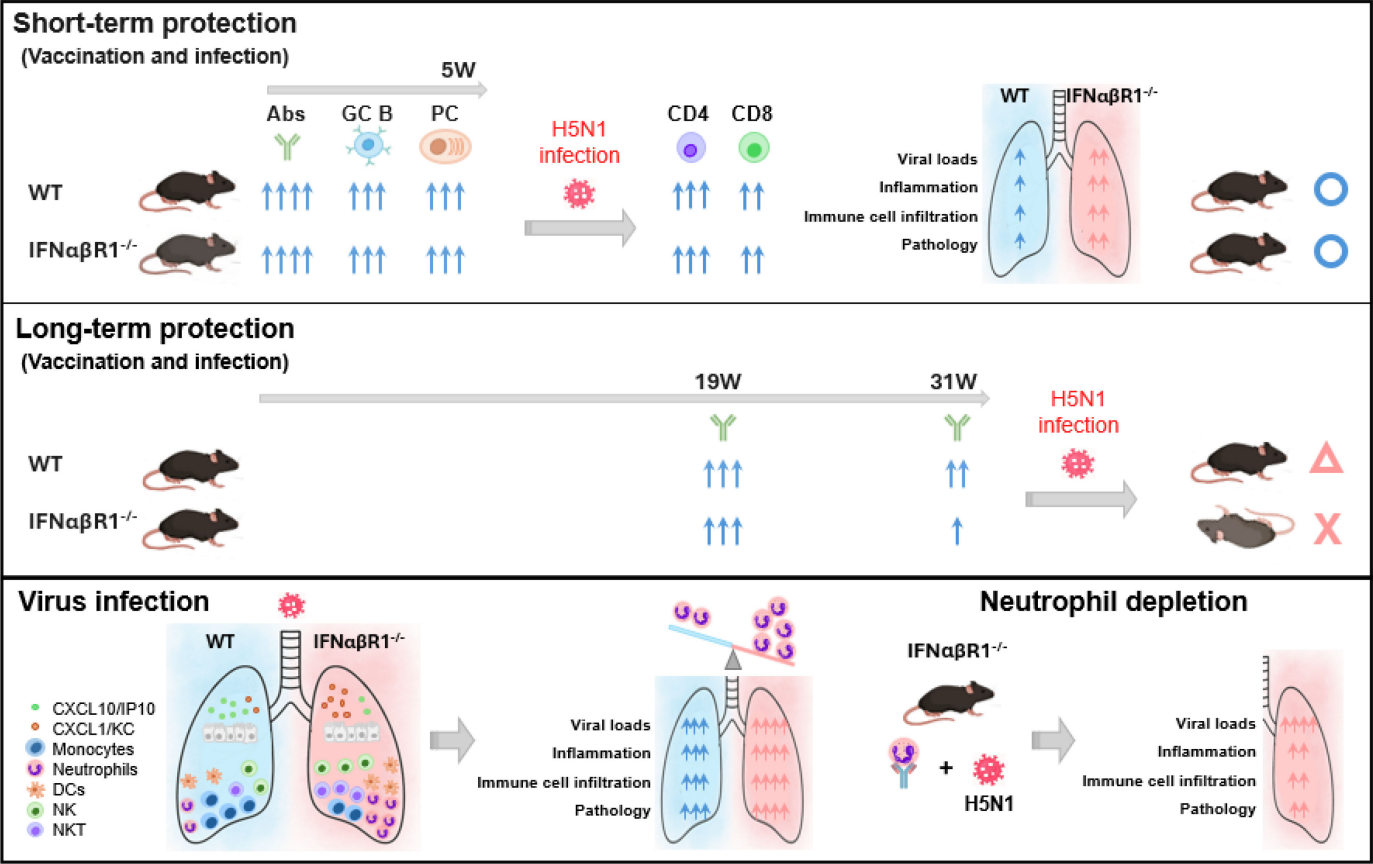
Schematic summary of the immune responses and protective effects in WT and IFNαβR1^-/-^ mice following H5 VLP vaccination and H5N1 virus challenge. Top panel: In the short term (5 weeks post-vaccination), both WT and IFNαβR1^-/-^ mice show robust antibody (Abs), germinal center B cell (GC B), and PC responses, leading to effective CD4^+^ and CD8^+^ T cell activation and protection against H5N1 infection. Middle panel: In the long term (31 weeks post-vaccination), WT mice maintain antibody responses and long-term protection at a moderate level, whereas IFNαβR1^-/-^ mice exhibit reduced antibody levels and diminished protection. Bottom panel: During H5N1 infection, IFNαβR1^-/-^ mice display increased viral loads, inflammation, and immune cell infiltration, particularly neutrophils. Neutrophil depletion in IFNαβR1^-/-^ mice alleviates lung pathology, indicating a key role for neutrophils in disease severity.

This study provides a comprehensive analysis of the role of type I IFNαβ receptor signaling in regulating host immune responses to influenza virus infection or vaccination, as shown in the graphic summary (Fig. 7). IFNAR-deficient (AB6) mice exhibited markedly increased susceptibility to influenza virus infection, even at sublethal doses, as evidenced by accelerated weight loss, elevated lung viral loads, severe histopathological damage, and increased mortality. These findings are consistent with previous reports that have established type I IFNs as essential mediators of early antiviral defense and viral clearance (19–21).

Despite impaired innate antiviral responses, AB6 mice mounted robust vaccine-induced short-term IgG antibodies. These responses included comparable HAI titers, plasma cell differentiation, and germinal center B cell formation at 5 weeks after boost, which collectively conferred adequate protection against lethal heterologous rgH5N1 challenge (Fig. 7). This indicates that short-term protective B cell responses can be induced independently of type I IFNαβ signaling, particularly under conditions of innate stimulation by immunogenic vaccines or virus infection, which is an observation consistent with previous reports (11, 22), demonstrating that mRNA or alphavirus replicon vaccines elicited robust virus-specific IgG and protective immunity in IFNAR-deficient mice. While AB6 mice generated early IgG responses efficiently in this study, IgG titers and protective immunity declined over time, with marked reductions at mid-term (19 weeks) and long-term (31 weeks) relative to wild-type B6 mice. These findings suggest that type I IFNαβR signaling is critical for maintaining IgG levels over time, although it is dispensable for the induction of early IgG responses following H5 VLP vaccination (Fig. 7).

Prior studies reported the importance of B-cell-intrinsic type I IFN signaling for B-cell activation, using different approaches (23, 24). Lower levels of IgG1 titers were reported in IFNAR^-/-^ mice than in B6 wild-type mice, at early time points (2–4 weeks) after immunization with NP-OVA plus poly I:C (a stimulator for type I IFN production) adjuvant, which continued to decline for 1 year at a comparable slope as in B6 mice (23). In particular, IFNAR^-/-^ mice showed early Th1-type IgG2c titers that were significantly lower than those of B6 mice at 2 weeks after immunization, and these low IgG2c titers persisted for 1 year (23). Significantly lower IgG levels were reported in IFNAR^-/-^ mice during early time points (at 20, 30 days) after immunization with T-independent type 2 polysaccharide antigens or Pneumovax23, probably due to a defect in B-1b cell-intrinsic type I IFNAR signaling, but long-term humoral responses and functional significance (impact on protective immunity) were not investigated (24). It is notable that the inclusion of adjuvant in Pneumovax23 immunization bypassed the requirement for IFNAR signaling (24). In contrast, this study found that type I IFN signaling was not required for the generation of early IgG antibody responses to H5 HA VLP vaccination or influenza virus infection. Importantly, several vaccine studies in IFNAR⁻^/^⁻ mice have reported the induction of neutralizing antibodies and survival protection upon viral challenge. In other studies, measles-based vaccines against Zika virus elicited protective antibody responses in IFNAR⁻^/^⁻ mice for several weeks after immunization (25). Subunit vaccines against Schmallenberg virus induced robust IgG responses and reduced disease severity in IFNAR-deficient mice (26). These findings support the notion that type I IFN signaling is not required to induce vaccine-mediated protective antibodies. Nonetheless, our findings suggest that primed B cells in AB6 mice might exhibit a defect in plasma cell longevity, but not in short-term IgG production in response to H5 VLP vaccination or virus infection. The mechanisms underlying why IgG wanes faster in AB6 mice, and the roles of IFNAR in long-lived plasma cells and memory B cells, remain to be addressed.

In this study, we identified an altered chemokine network in vaccinated and unvaccinated mice lacking IFNαβR signaling after influenza virus infection. AB6 mice exhibited increased levels of CXCL1/KC, which might have been linked to high mobilization of neutrophils in AB6 mice. In contrast, AB6 mice showed reduced levels of CXCL10/IP-10, resulting in impaired trafficking of monocytes and DCs. AB6 mice exhibited reduced levels of activated DCs in airway BAL fluids, in contrast to the accumulation in lung tissues, suggesting that type I IFNαβR signaling plays a role in the spatial coordination of DC mobilization. Indeed, IFN-induced chemokine gradients and receptor expression, such as CXCL10 and CCR7, might regulate DC migration from peripheral tissues into lymphoid compartments and the airway surfaces. This is consistent with previous reports that type I IFN impacts DC subset localization by modulating their responsiveness to migratory cues, effectively shaping their tissue compartmentalization during influenza virus infection (5, 27, 28). This altered APC landscape may influence both T cell priming and immunopathology. Heightened infiltration of CD4^+^ and CD8^+^ effector T cells, NK/NKT cells, and activated macrophages was observed in AB6 mice, accompanied by exacerbated neutrophil recruitment and pulmonary inflammation. Additionally, the exaggerated recruitment of myeloid (neutrophils, DCs, macrophages) and lymphoid populations (NK, NKT, T cells) to the lung after influenza virus infection or peritoneal exudates of the vaccine injection site in AB6 mice suggests that IFNαβR signaling also modulates early innate cell mobilization following immunization or virus infection (Fig. 7).

Neutrophils, as a key effector cell of the innate immune system, play a dual role during influenza virus infection, mediating both protective and pathogenic responses. On the other hand, excessive or prolonged neutrophil activation has been shown to drive tissue injury, pulmonary vascular leakage, and exacerbated immunopathology (12, 13). Our findings highlight the pivotal role of type I IFNαβR signaling in modulating early neutrophil recruitment during vaccination and subsequent viral challenge (Fig. 7). In IFNαβR-deficient AB6 mice, we observed an accumulation of neutrophils at the site of vaccine injection and in the lungs of vaccinated or naïve mice during influenza virus infection. This was accompanied by a shift in myeloid cell composition, with a reduction in monocyte infiltration, indicating that type I IFN signaling orchestrates granulocyte-monocyte balance during acute inflammation. Previous studies reported that early monocyte recruitment to the site of immunization supports antigen uptake, antigen presentation, and efficient T cell priming (29, 30). In contrast, excessive or prolonged neutrophil accumulation has been shown to negatively regulate adaptive immune responses by limiting antigen availability, suppressing dendritic cell function, or promoting a highly inflammatory milieu (31, 32). The neutrophil-dominant, monocyte-deficient innate immune environment observed in AB6 mice on day 1 post-injection might reflect an early immune landscape less favorable to the longevity of vaccine-induced adaptive immunity. This insight might provide a new framework for understanding how altered early innate responses may contribute to long-term immunity downstream of vaccination.

Antibody-mediated neutrophil depletion alleviated weight loss and lung pathology without altering viral titers. These results support a model in which neutrophil-driven immunopathology alters immune cell infiltrations and contributes to disease, alongside higher viral loads in the absence of IFNAR signaling. These findings are consistent with previous studies showing that neutrophils can exacerbate immunopathology during influenza virus infection (12, 13). Moreover, our data demonstrate that neutrophils influence the recruitment of DCs, including CD11b^+^ DCs, and shape downstream adaptive immunity. Neutrophil depletion led to reduced numbers of CD8^+^ or CD69^+^CD8^+^ T cells, CD4^+^ or CD69^+^CD4^+^ T cells, and NK/NKT or CD69^+^NK/NKT cells, indicating that infiltration of neutrophils could modulate innate and adaptive immunity in the IFNαβR-deficient setting. Taken together, this study suggests that type I IFNαβR signaling restrains excessive neutrophil-driven inflammation by regulating chemokine expression and cellular trafficking during immune activation. The absence of this regulatory axis of IFNαβR signaling results in a hyper-inflammatory milieu dominated by granulocytic infiltration, contributing to immunopathology (Fig. 7).

In summary, this study demonstrates that type I IFNαβR signaling serves as a multifaceted and indispensable regulator of antiviral immunity during influenza vaccination and virus infection (Fig. 7). Although it is not essential for the initial induction of humoral responses, IFNαβR signaling is crucial for long-term maintenance of IgG antibodies and sustained protective immunity. At the cellular level, it coordinates immune cell trafficking and distribution in the tissue, maintaining balanced recruitment of granulocytes, monocytes, and dendritic cells. Disruption of this signaling pathway leads to chemokine imbalance, excessive neutrophil infiltration, aberrant DC migration, and heightened pulmonary inflammation. Collectively, these findings support a central immunoregulatory axis of type I IFNαβR signaling that integrates innate and adaptive immune responses to promote antiviral defense.

## MATERIALS AND METHODS

### Mice

Wild-type C57BL/6 (B6) mice were purchased from Jackson Laboratory. Type I interferon receptor-deficient (IFNαβR⁻/⁻; AB6) mice on a C57BL/6 background (33), which were first reported to have antiviral roles for IFNαβR, were generously provided by Dr. Sujan Shresta (La Jolla Institute for Allergy & Immunology, La Jolla, CA) and bred in-house.

### Viruses and vaccines

Recombinant reassortant H5N1 influenza viruses (rgH5N1) were produced via reverse genetics (rg), incorporating HA and neuraminidase genes from A/Vietnam/1203/2004 (H5N1) or the HA of A/Indonesia/05/2005 (H5N1), while the internal genes were derived from the A/Puerto Rico/8/1934 (H1N1) backbone, as previously described (34, 35). Influenza A viruses were propagated in 10-day-old embryonated chicken eggs and subsequently inactivated by treatment with 0.01% formalin for 72 h. Residual formalin was removed by dialysis, and inactivated virus stocks were stored at −80°C until use for ELISA coating antigens. Influenza H5 VLPs, consisting of the HA protein from A/Indonesia/05/2005 and the matrix protein M1 from A/PR8/34, were produced in Sf9 insect cells using the Bac-to-Bac baculovirus expression system (Invitrogen), following established protocols (34).

### Vaccination and challenge of mice

Female C57BL/6 (B6) and type I interferon receptor-deficient (IFNαβR^-/-^) B6 background (AB6) mice, aged 6–8 weeks, were immunized intramuscularly twice with 10 µg of H5 VLP in a total volume of 100 µL (50 µL per hind leg) at a 3 week interval. To evaluate the durability of cross-protective immunity, immunized mice were challenged intranasally with a heterologous A/Vietnam/1203/2004 (rgH5N1) virus at three distinct time points post-boost: short-term (5 weeks), mid-term (19 weeks), and long-term (31 weeks). Mice with or without vaccination were anesthetized using isoflurane and inoculated with a sublethal dose (0.2–0.8×LD₅₀) and a lethal dose (2×LD₅₀) of virus in 50 µL PBS. Following the challenge, body weight and survival rates were monitored daily for 14 days. Survival data were plotted as Kaplan-Meier survival curves and statistically analyzed using the log-rank (Mantel-Cox) test. Mice that reached the humane endpoint, defined as 20%–25% loss of body weight or severe illness accompanied by loss of activity, were euthanized in accordance with institutional animal welfare guidelines.

### Assessment of antibody and chemokine responses

Serum antibody responses were evaluated as previously described (36). Briefly, sera collected at 5 weeks, 19 weeks, and 31 weeks post-boost were serially diluted in PBS with 0.05% Tween 20 (PBST) and added to 96-well plates pre-coated with 2 µg/mL of inactivated whole-virus antigen (rgH5N1; Indonesia or Vietnam strain). ELISA plates were incubated at room temperature (RT), and bound antibodies were detected using horseradish peroxidase-conjugated goat anti-mouse IgG, IgG1, and IgG2c secondary antibodies (Southern Biotech, Birmingham, AL, USA). Color development was achieved using 3,3′,5,5′-tetramethylbenzidine (eBioscience, San Diego, CA, USA), and the reaction was terminated with 0.5 M sulfuric acid (H_2_SO_4_). Optical density (OD) was measured at 450 nm across serial dilutions using a microplate reader (Bio-Rad, Hercules, CA, USA). The overall magnitude of the dilution response was quantified by calculating the AUC of the absorbance versus log (dilution) plot, using a cut-off value equal to the mean absorbance of blank wells. This approach provides an integrated measure of signal across all dilutions (37, 38). Inflammatory chemokines (CXCL-1/KC and CXCL-10/IP-10) were measured using DuoSet ELISA kits (R&D Systems, Minneapolis, MN, USA) according to the manufacturer’s instructions. Chemokine levels were analyzed from lung tissue lysates and BALF samples collected on day 5 post-challenge. For comprehensive visualization, chemokine data were also analyzed using heat map representations (GraphPad Prism 10.0), with values normalized across samples to illustrate relative expression patterns in BAL and lung tissues.

### HAI assay

HAI titers were measured in sera collected after booster immunization, following established protocols (36). Briefly, sera were treated with receptor-destroying enzyme (RDE II; Denka Co., Ltd., Tokyo, Japan) overnight at 37°C, followed by heat inactivation at 56°C for 30 min. Twofold serial dilutions of the treated sera were prepared in PBS and incubated with eight HA units of reassortant rgH5N1 viruses (A/Vietnam/1203/2004, A/Indonesia/05/2005) for 1 h at RT. Following incubation, 0.5% chicken red blood cells (RBCs; Lampire Biological Laboratories, Pipersville, PA, USA) were added to each well. Plates were incubated at RT until RBC precipitation was observed. HAI titers were defined as the highest serum dilution that completely inhibited hemagglutination.

### Virus titration

To quantify viral replication, lung tissues were harvested from mice on day 5 post-challenge with reassortant A/Vietnam/1203/2004 (rgH5N1) virus. Lungs were homogenized using a sterile strainer (70 µm), and the homogenates were serially diluted 10-fold with 1× PBS. Each dilution was inoculated into the allantoic cavity of 10-day-old embryonated chicken eggs (Hy-Line North America, LLC., Mansfield, GA, USA), followed by incubation at 37°C for 72 h. Allantoic fluids were then harvested, and hemagglutination activity was assessed as an indicator of the presence of live viruses. Viral titers were calculated using the Reed and Muench method (39) and expressed as the EID₅₀ per lung.

### Flow cytometry

Single-cell suspensions were prepared from MLNs obtained post-vaccination and challenge to assess effective B cell responses. Tissues were passed through a 70 µm nylon mesh and resuspended in PBS containing 0.5% fetal bovine serum and 1 mM EDTA. Cells were first incubated with anti-CD16/32 (Fc block; clone 14-0161-85, eBioscience) and stained with surface antibodies against CD3 (clone 17A2, BioLegend), CD19 (clone eBio1D3, eBioscience), IgD (clone 11.26, eBioscience), B220 (clone RA3-6B2, BD Pharmingen), CD138 (clone 281-2, BD), and GL7 (clone GL7, eBioscience). Plasma cells and germinal center B cells were identified by flow cytometry and gated as described in Fig. S6.

The lungs were perfused (to collect BAL fractions), mechanically dissociated, and a fraction was saved for histopathology. Half of the lung tissues were used to prepare cellular fractions for flow cytometry and to extract supernatants for plaque assays. To evaluate innate and adaptive immune cell responses, single-cell suspensions were also prepared from BALF and lung tissue at day 5 or 6 post-challenge, as previously described (40). For intracellular cytokine staining, single-cell suspensions were stimulated *in vitro* with 4 µg/mL of inactivated rgH5N1 (Indonesia strain) in the presence of brefeldin A (20 µg/mL) for 5 h at 37°C. After surface staining with antibodies against CD4 (clone RM4-5, eBioscience) and CD8 (clone 53-6.7, eBioscience), cells were fixed and permeabilized using the BD Cytofix/Cytoperm Plus kit (BD Biosciences), followed by intracellular staining with anti-mouse IFN-γ (clone XMG1.2, eBioscience) and anti-mouseTNF-α (clone MP6-XT22, BioLegend). Effector T cells were gated as shown in Fig. S2.

For innate immune cell profiling, single-cell suspensions were prepared from BALF, lung tissues, and peritoneal exudates. BALF was collected by flushing the airways with cold PBS (0.6 mL × 2 times) through the trachea using a catheter (18G×1_1/4_”, EXEL^INT^). Peritoneal cells were isolated by injecting cold PBS (1 mL × 2 times) into the peritoneal cavity. Followed by gentle massage and aspiration of the peritoneal fluid. The collected fluids were centrifuged, and cell pellets were resuspended in FACS buffer for downstream staining. The single-cell suspensions were stained with monoclonal antibodies specific for mouse CD11b (clone M1/70, BD), CD11c (clone N418, eBioscience), F4/80 (clone BM8, eBioscience), Ly6C (clone HK1.4, BioLegend), Siglec-F (clone E50-2440, BD), MHC II (clone M5/114.15.2, BioLegend), CD103 (clone 2E7, BioLegend), CD3 (clone 17A2, BioLegend), CD49b (clone DX5, BioLegend), and CD69 (clone H1.2F3, eBioscience). CD16/32 (clone 93, eBioscience) was used for blocking Fc receptors. Flow cytometry analysis was performed on a BD Fortessa flow cytometer, and data acquisition was conducted with FACSDiva software (BD Biosciences). Gating strategies for immune cell subsets are presented in Fig. S4. Data were analyzed using FlowJo software version 10.10 (Tree Star, Ashland, OR, USA).

### Histopathological analysis

To assess pulmonary inflammation, vaccinated and virus-challenged B6 and AB6 mice, as well as neutrophil-depleted AB6 mice, were sacrificed at designated time points post-infection. Lungs were fixed in 10% neutral-buffered formalin for 48 h. Tissues were paraffin-embedded, sectioned at 5 µm, and stained with H&E. Histopathological evaluation was performed in a blinded manner using light microscopy. Inflammatory severity was scored semi-quantitatively (0–3 scale) based on immune cell infiltration in three anatomical regions: airway, blood vessel (perivascular), and interstitial space. The scoring criteria were as follows: 0 indicates no inflammation, 1 indicates mild (few loosely arranged cells), 2 indicates moderate pathology (many cells in the peripheral parts of the perivascular space), and 3 indicates severe (numerous cells in the perivascular space) inflammation (41, 42). Representative images were captured using a bright-field microscope equipped with a digital imaging system.

### *In vivo* neutrophil depletion

To investigate the role of neutrophils during influenza virus infection, *in vivo* neutrophil depletion was performed in AB6 mice using a mAb targeting Ly6G (43). Mice were intraperitoneally injected with 400 µg of anti-mouse Ly6G mAb (clone 1A8; Bio X Cell, Lebanon, NH, USA) or PBS as a control on days 0 and +2, either prior to or following infection. The efficiency of neutrophil depletion and its impact on other innate immune cell populations, as shown in Fig. S4, were assessed by flow cytometric analysis of single-cell suspensions prepared from lung tissue collected at day 6 post-infection.

### Statistical analysis

All quantitative data are presented as the mean ± SEM. Comparisons among groups were conducted using two-way ANOVA followed by multiple comparisons tests, including Tukey’s multiple comparisons. Paired *t*-tests were used for comparisons between two related groups. Survival rates of mice were analyzed using the log-rank (Mantel-Cox) test. A *P*-value <0.05 was considered statistically significant. All analyses were performed using GraphPad Prism 10.0 (GraphPad Software, San Diego, CA, USA).

## SUPPLEMENTAL MATERIAL

Supplemental figures jvi.00229-26-s0001.pdf Figures S1 to S7.

ASM does not own the copyrights to Supplemental Material that may be linked to, or accessed through, an article. The authors have granted ASM a non-exclusive, world-wide license to publish the Supplemental Material files. Please contact the corresponding author directly for reuse.
