# Supplementary material for: Type I interferon signaling is required for resistance to primary influenza virus infection and vaccine-induced long-term immunity
Source: J Virol. 2026 Mar 27;100(4):e00229-26. doi: 10.1128/jvi.00229-26 (PMC13098210; doi:10.1128/jvi.00229-26)
Supplement: Supplemental figures — Figures S1 to S7. [file jvi.00229-26-s0001.pdf]

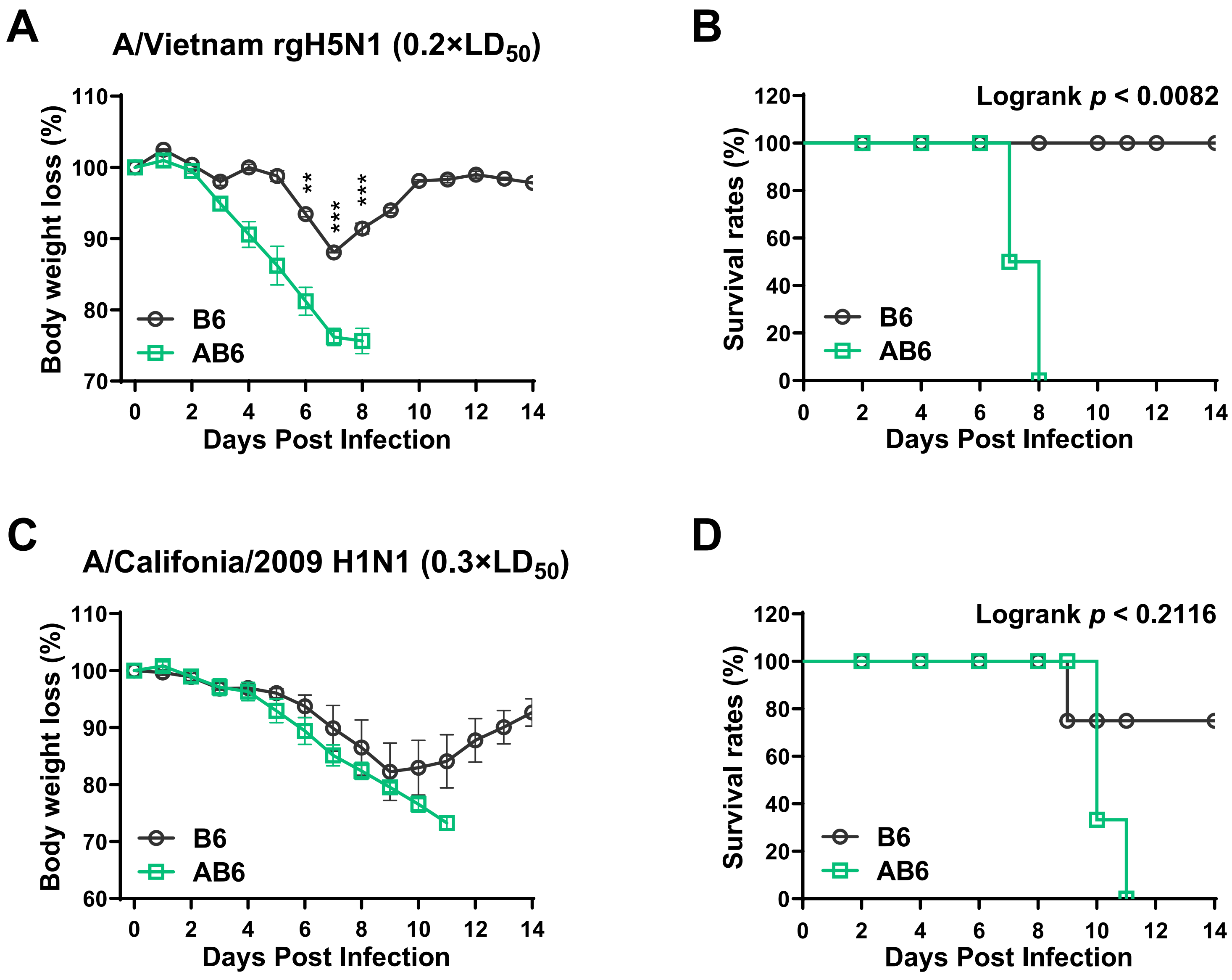

Supplementary Fig. S1. Enhanced susceptibility of IFNαβR-deficient mice to influenza infection.

Naïve AB6 (IFNαβR$^{-/-}$) and C57BL/6 (B6) mice (n=3-4 mice per group) were intranasally infected with influenza viruses. Body weight changes and survival rates were monitored daily for 14 days. (**A, B**) Body weight changes and survival of B6 and AB6 mice following sublethal A/Vietnam rgH5N1 infection (0.2×LD_{50}). (**C, D**) Body weight changes and survival following sublethal A/California/2009 H1N1 infection (0.3×LD_{50}). Data are presented as mean ± SEM. Statistical analysis was performed using two-way ANOVA with multiple comparisons test and survival curve comparison with the Log-rank (Mantel-Cox) test. **, p<0.01; ***, p<0.001.

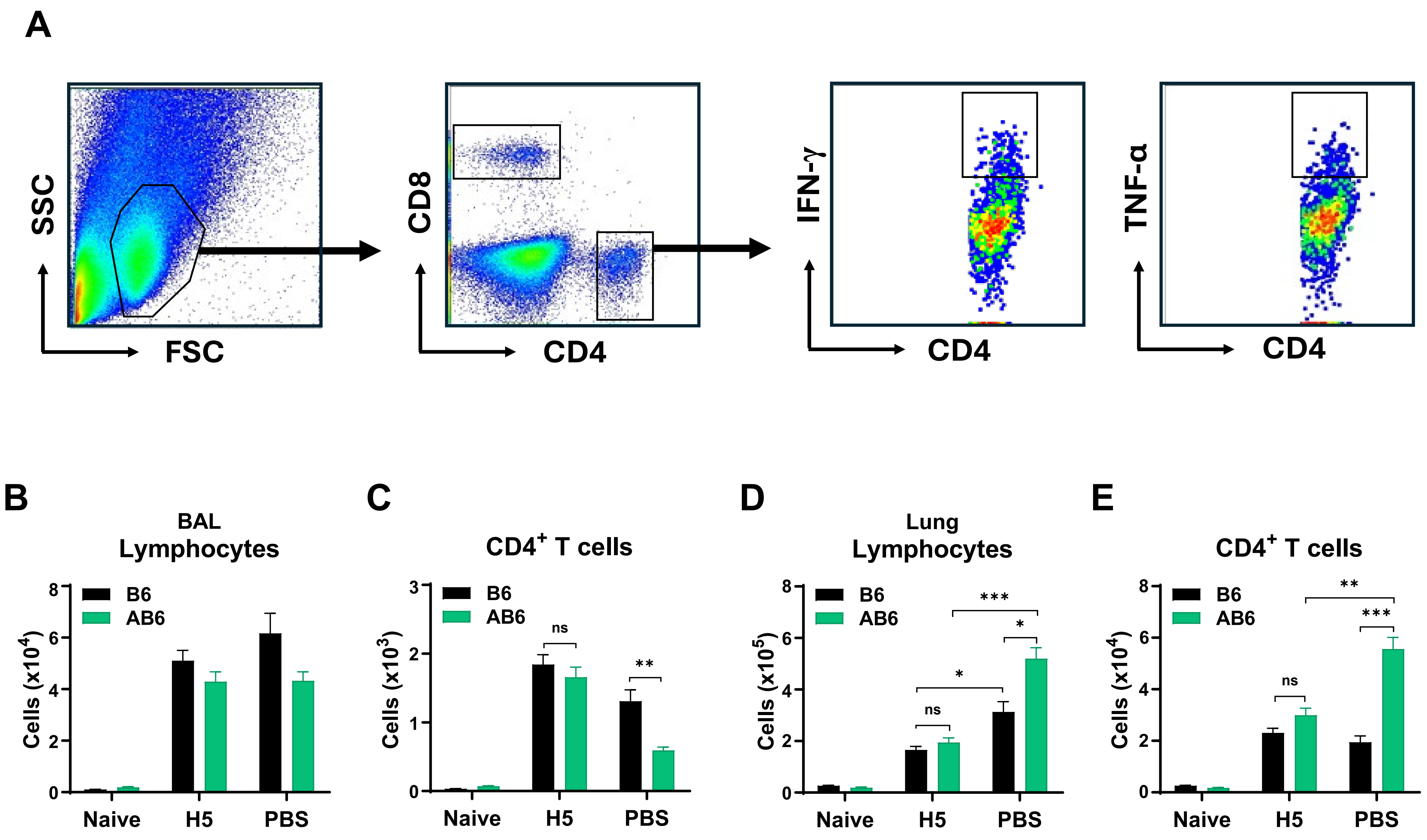

Supplementary Figure S2. Pulmonary T cell responses following influenza virus infection. (A) Representative flow cytometry gating strategy for identification of CD4$^+$ and CD8$^+$ T cells and their intracellular cytokine expression. Lymphocytes were gated based on FSC/SSC, followed by CD4 and CD8 surface staining. Intracellular IFN-γ and TNF-α production was analyzed in CD4$^+$ cells after stimulation. **(B-E)** Total number of lymphocytes and CD4$^+$ T cells in bronchoalveolar lavage (BAL) fluid **(B, C)** and in lung tissues from B6 and AB6 mice at day 5 post-infection with A/Vietnam rgH5N1 virus. Data are represented as mean ± SEM. Statistical significance was determined by two-way ANOVA with Tukey's multiple comparisons. *, p<0.05; **, p<0.01, **, p<0.001, ns: not significant.

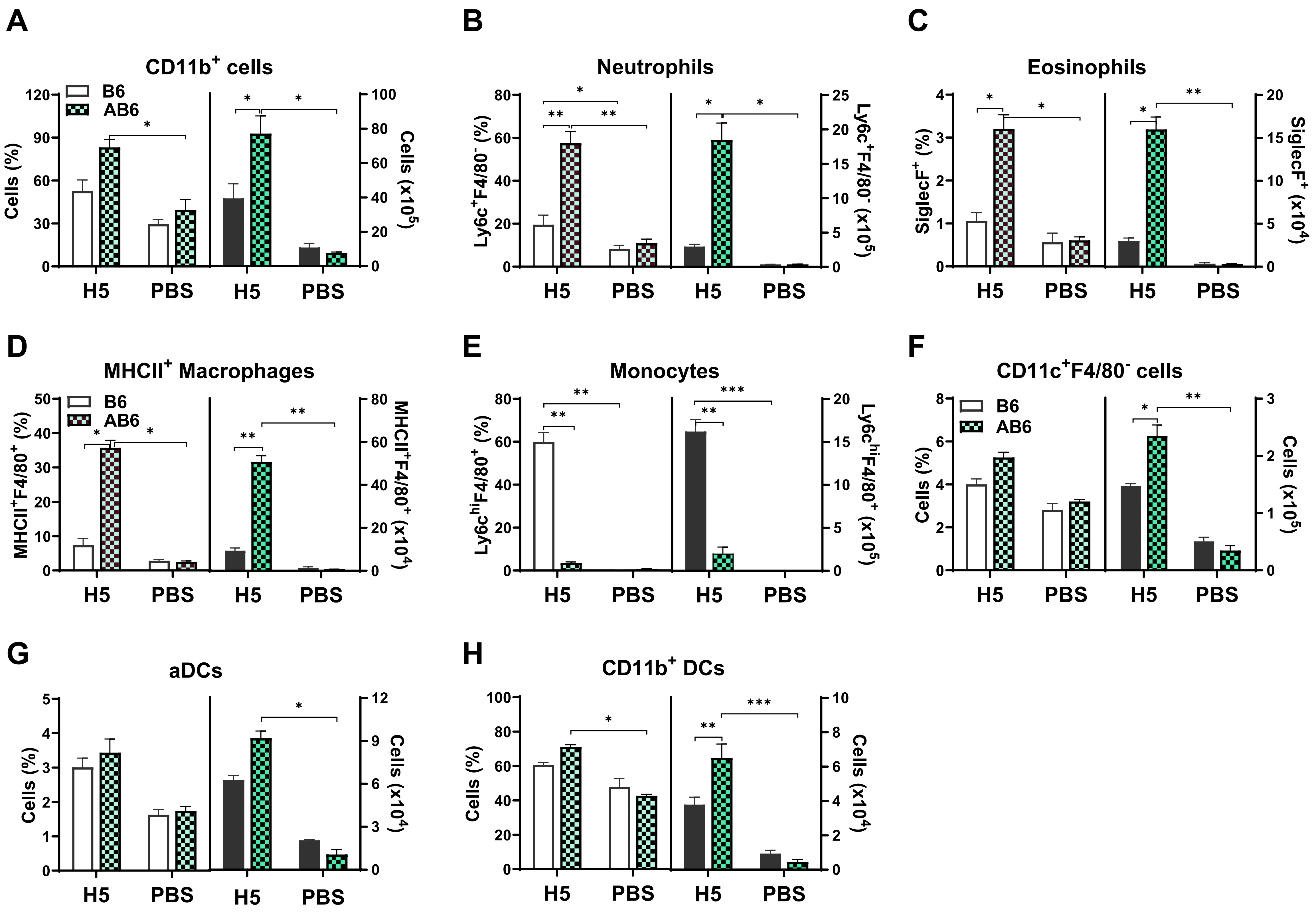

Supplementary Figure S3. AB6 mice exhibit enhanced recruitment of innate immune cells at the vaccine injection site within a day. Naïve AB6 and B6 mice (n=3 or 4 mice per group) were intraperitoneally (IP) injected with 10 µg of H5 HA VLP (H5) vaccine or PBS (mock). Peritoneal exudate cells were collected 24 hours post-injection and analyzed by flow cytometry. **(A)** $CD11b^+$ (percentages, cell numbers) out of total myeloid cells were identified after exclusion of debris and lymphocytes based on forward and side scatter gating. **(B-E)** Frequencies and absolute numbers of neutrophils ($Ly6c^+F4/80^-$), eosinophils ($SiglecF^+CD11c^-$), macrophages ($MHCII^+F4/80^+CD11c^-$), and monocytes ($Ly6c^{hi}F4/80^+$) gated from the $CD11b^+$ myeloid cell population. Percentages shown on the y-axis represent the proportion of each indicated population within the $CD11b^+$ gate. **(F-H)** Frequencies and numbers of $CD11c^+F4/80^-$ cells, activated DCs (aDCs; $CD11c^+MHCII^+F4/80^-$), and $CD11b^+$ DCs ($CD11b^+CD11c^+MHCII^+F4/80^-$). Data are represented as mean ± SEM. Statistical analysis was performed using two-way ANOVA with Tukey's multiple comparisons test. *, $p < 0.05$; **, $p < 0.01$; ***, $p < 0.001$.

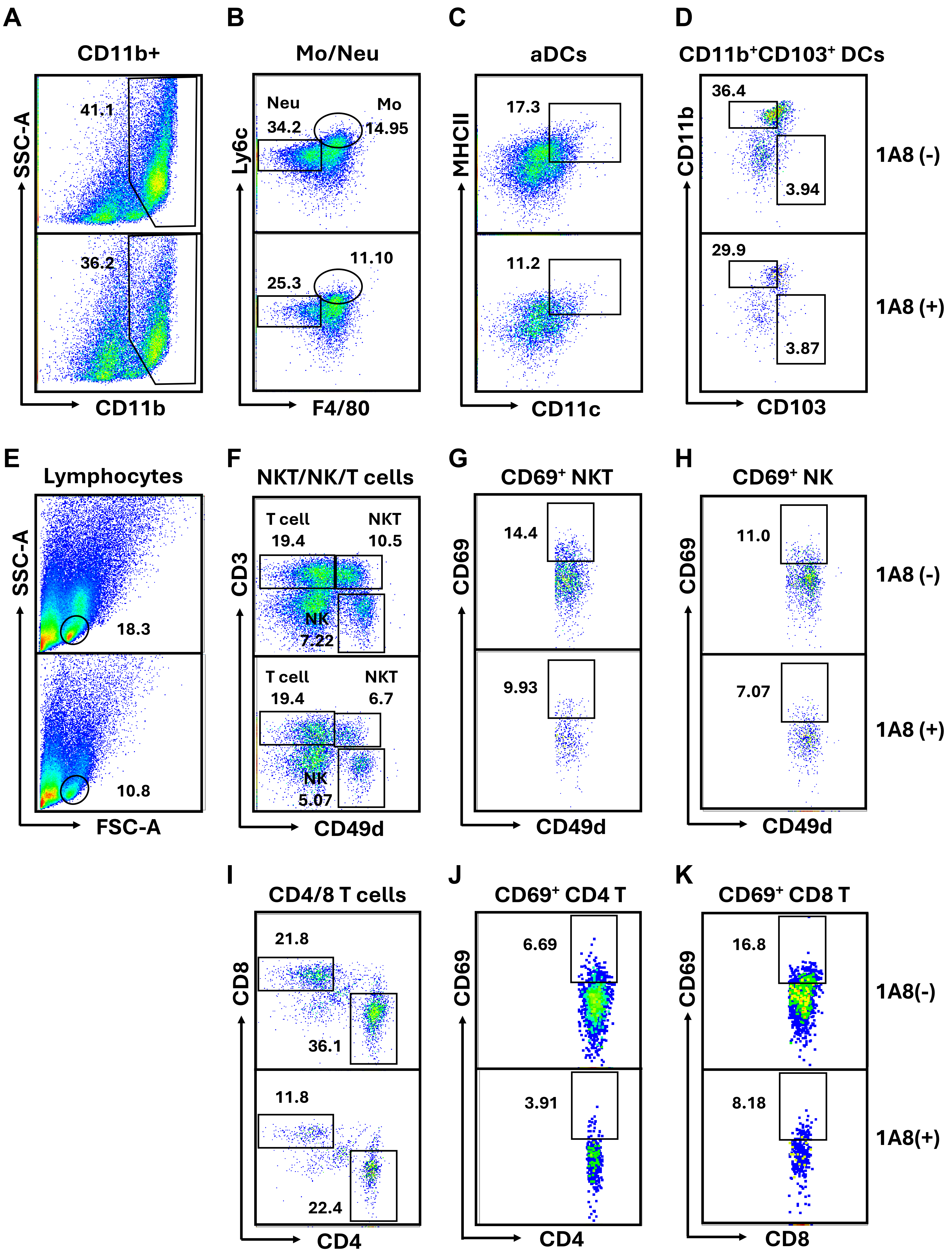

Supplementary Figure S4. Flow cytometric analysis of myeloid and lymphoid cell populations following neutrophil depletion. Single-cell suspensions were prepared from the lung tissues of AB6 mice +/- anti-Ly6G antibody (1A8 mAb) treatment and analyzed by flow cytometry. **(A)** Gating of CD11b^{+} myeloid cells from live singlet populations. **(B)** Identification of monocytes and neutrophils (Mo/Neu) based on Ly6c and F4/80 expression within the CD11b^{+} gate. **(C)** Activated dendritic cells (aDCs) defined as MHCII^{+}CD11c^{+} cells. **(D)** CD11b^{+} and CD103^{+} DC subsets gated within the CD11c^{+}MHCII^{+} population. **(E)** Gating strategy for lymphocytes based on forward scatter (FSC-A) and side scatter (SSC-A). **(F)** Identification of T cells (CD3^{+}CD49d^{-}), NKT cells (CD3^{+}CD49d^{+}), and NK cells (CD3^{-}CD49d^{+}) within the lymphocyte gate. **(G)** Activated NKT cells as CD69^{+} NKT cells. **(H)** Activated NK cells as CD69^{+} NK cells. **(I)** Identification of CD4^{+} and CD8^{+} T cell subsets within CD3^{+} T cells. **(J)** Activated CD4^{+} T cells as CD69^{+}CD4^{+} T cells. **(K)** Activated CD8^{+} T cells as CD69^{+}CD8^{+} T cells. Representative flow cytometry plots from 1A8(-) (upper panels) and 1A8(+) (lower panels) AB6 mice are shown. Numbers indicate the percentage of cells within the indicated gates.

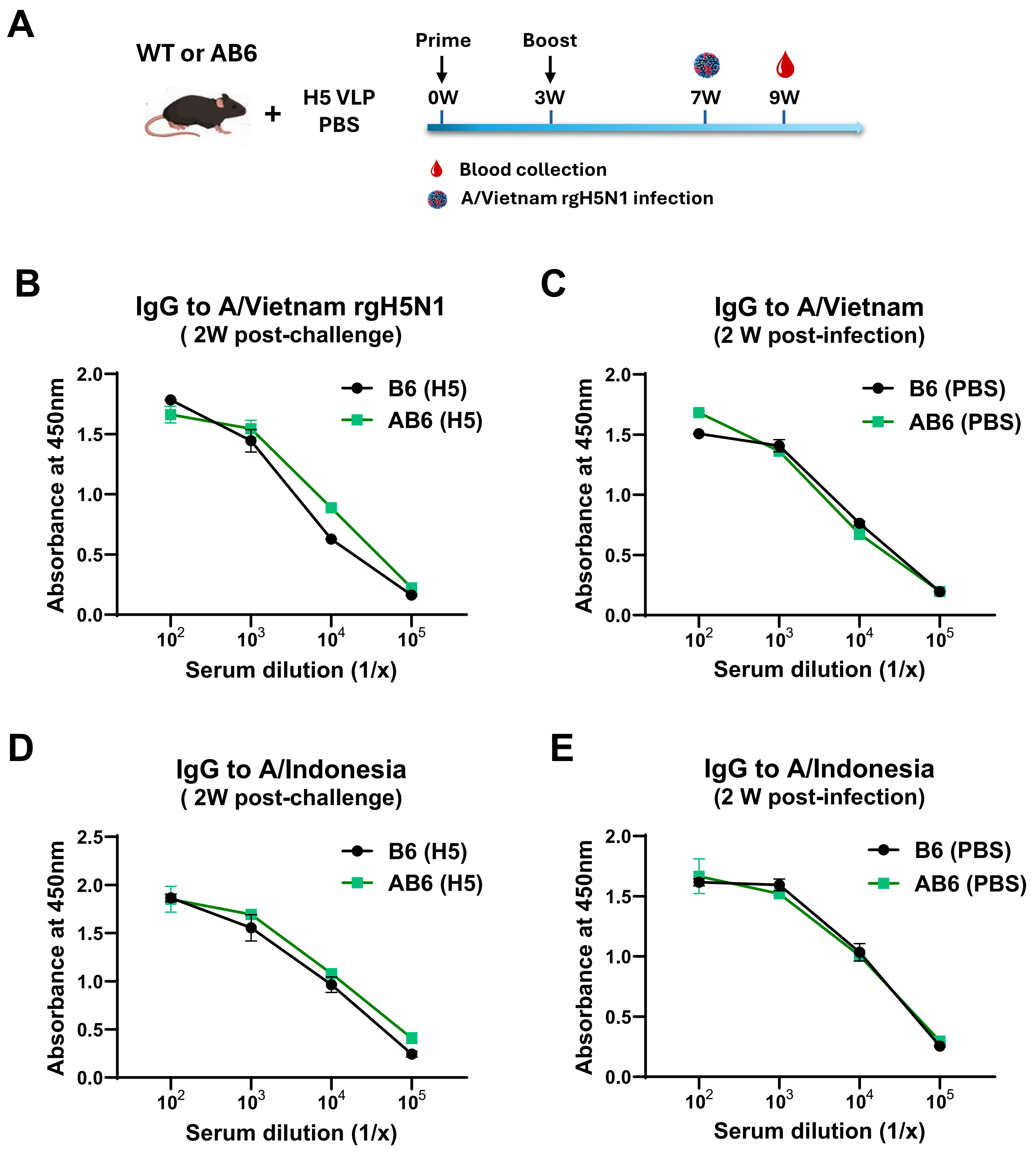

Supplementary Figure S5. Similar levels of antibodies are induced at 2-week time point after influenza virus infection. (A) Experimental design. AB6 and B6 (n=4-5 mice per group) were immunized with 10μg of H5 HA VLPs or PBS (no vaccine, mock control) and challenged with a lethal dose of A/Vietnam rgH5N1 virus at 7 weeks. Blood samples were collected at 2 weeks post-challenge for serological analysis. Antibody responses to A/Vietnam **(B, C)** or A/Indonesia **(D, E)** viral antigens in sera collected at 2 weeks after virus infection.

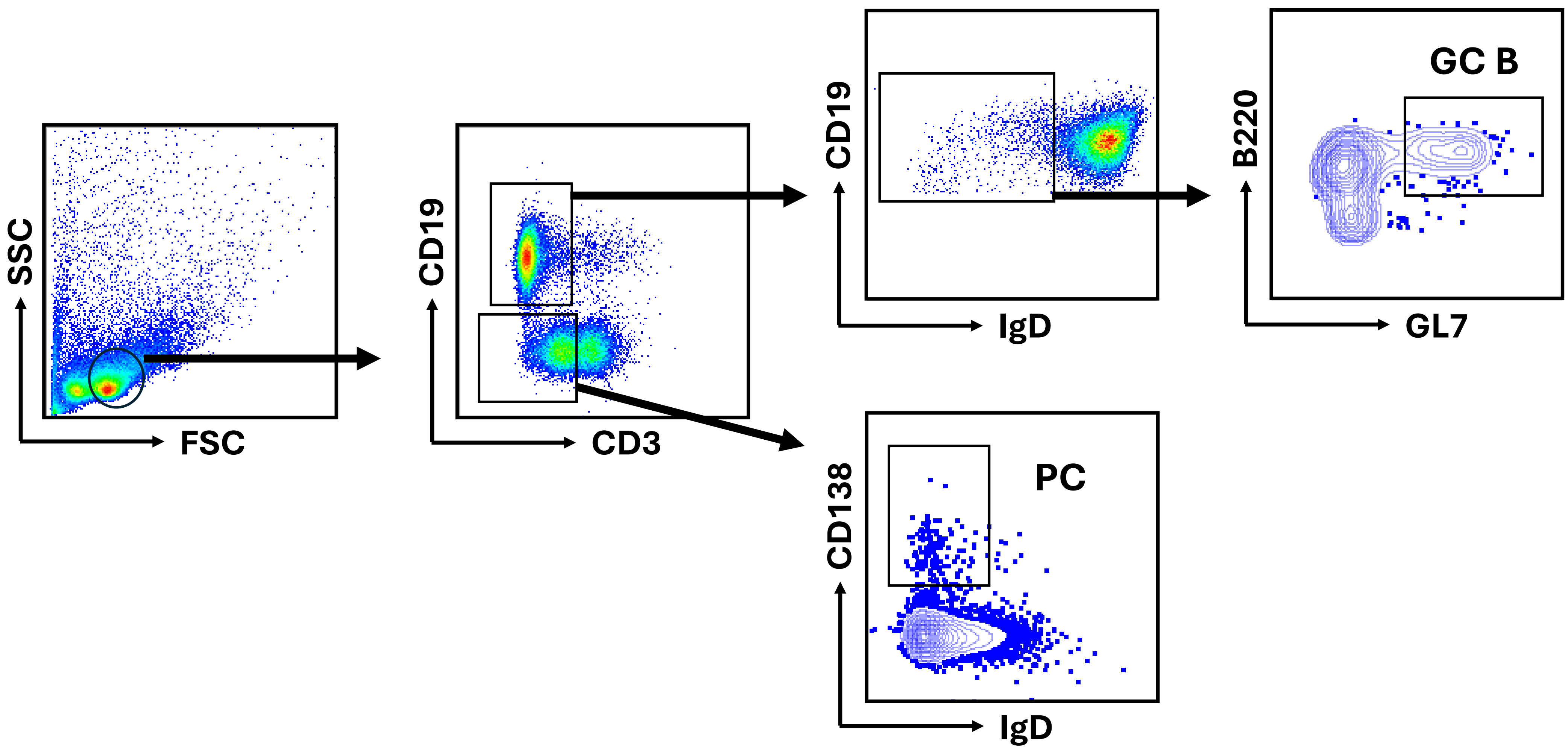

Supplementary Figure S6. Gating strategy for identification of germinal center B cells and plasma cells in mouse lymphoid tissues. Single-cell suspensions from lymphoid tissues were first gated based on forward scatter (FSC) and side scatter (SSC) to exclude debris and select lymphocyte populations. T cell were excluded by gating on $CD3^-$ cells, and B cells were identified as $CD19^+$ cells. Naïve B cells were excluded based on IgD expression, and $CD19^+IgD^-$ cells were further analyzed. Germinal center B (GC B) cells were defined as $CD19^+IgD^-GL7^+B220^+$ cells. Plasma cells (PCs) were identified as $CD3^-CD19^-IgD^-CD138^+$. Representative flow cytometry plots illustrating each gating step are shown.

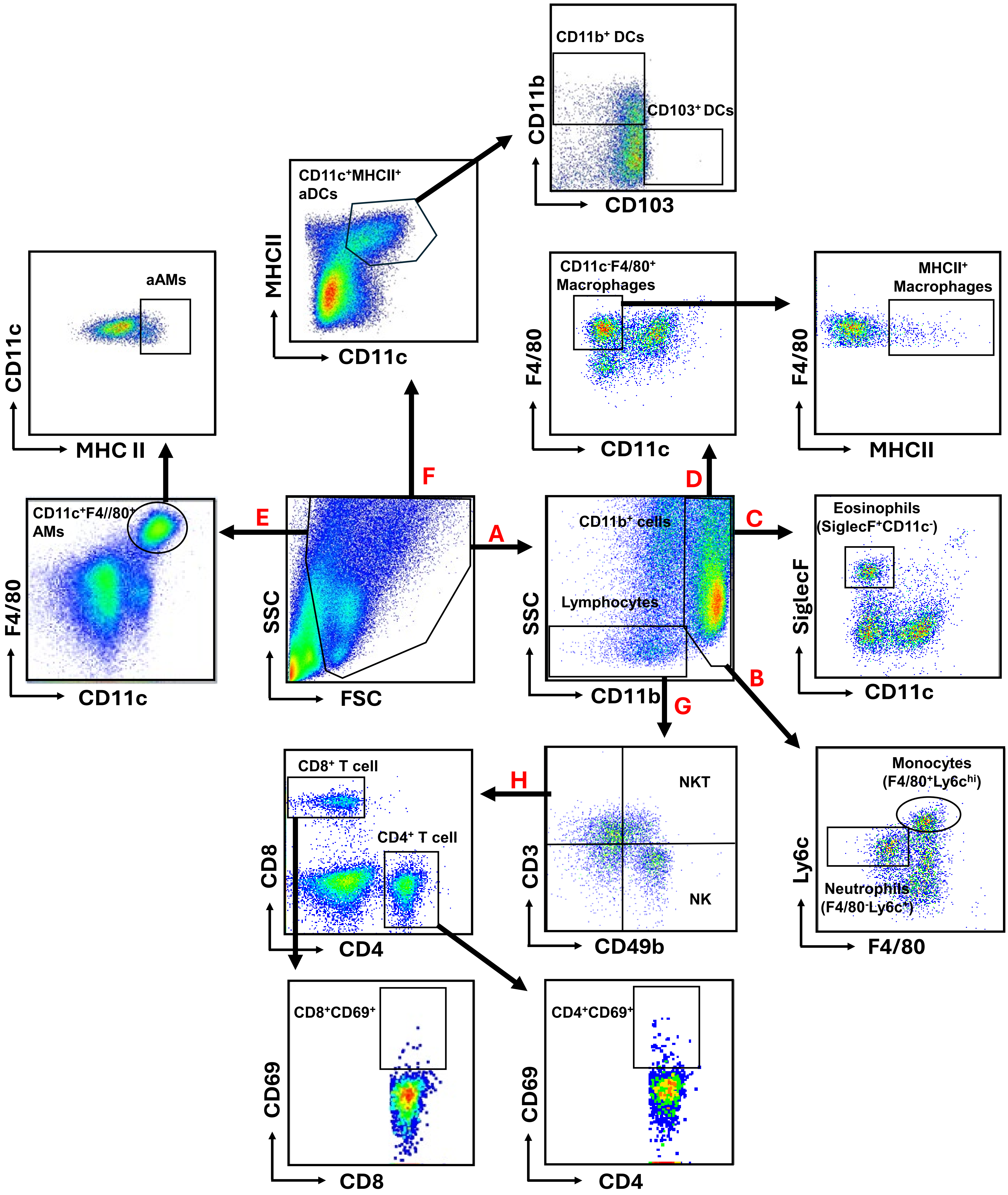

Supplementary Figure S7. Gating strategy for identification of innate and cellular immune cell subsets. Single-cell suspensions from tissue were analyzed by flow cytometry. **(A)** Initial gating was performed on lymphoid and myeloid populations based on CD11b expression, following pre-gating on singlets and live cells using forward scatter (FSC) and side scatter (SSC). **(B-D)** CD11b^{+} cells were further analyzed to identify neutrophils (CD11b^{+}Ly6C^{+}F4/80^{-}), monocytes (CD11b^{+}Ly6C^{hi}F4/80^{+}), eosinophils (CD11b^{+}Siglec-F^{+}CD11c^{-}), macrophages (CD11c^{-}F4/80^{+}), and activated macrophages (CD11c^{-}F4/80^{+}MHCII^{+}). **(E)** Alveolar macrophages (AMs) were defined as CD11c^{+}F4/80^{+}, with activated AMs (aAMs) identified by MHC II expression. **(F)** Dendritic cells (DCs), defined as CD11c^{+}MHC II^{+} cells (activated DCs; aDCs), were gated based on forward scatter (FSC-A) and side scatter (SSC-A) and further subdivided into CD11b^{+} DCs and CD103^{+} DCs within the CD11c^{+}MHCII^{+} population. **(G)** Natural killer (NK) and NKT cells were defined as CD3^{-}CD49b^{+} and CD3^{+}CD49b^{+}, respectively. **(H)** CD4^{+} and CD8^{+} T cells were identified within the CD3^{+}CD49b^{-} population, and activation status was assessed by CD69 expression (CD4^{+}CD69^{+} and CD8^{+}CD69^{+}).
